# Observing the overall rocking motion of a protein in a crystal

**DOI:** 10.1038/ncomms9361

**Published:** 2015-10-05

**Authors:** Peixiang Ma, Yi Xue, Nicolas Coquelle, Jens D. Haller, Tairan Yuwen, Isabel Ayala, Oleg Mikhailovskii, Dieter Willbold, Jacques-Philippe Colletier, Nikolai R. Skrynnikov, Paul Schanda

**Affiliations:** 1 Université Grenoble Alpes, IBS, F-38044 Grenoble, France; 2 CEA, Institut de Biologie Structurale, F-38044 Grenoble, France; 3 CNRS, Institut de Biologie Structurale, F-38044 Grenoble, France; 4 Department of Chemistry, Purdue University, West Lafayette, Indiana 47907, USA; 5 Institut für Physikalische Biologie, Heinrich-Heine-Universität Düsseldorf, 40225 Düsseldorf, Germany; 6 ICS-6: Structural Biochemistry, Forschungszentrum Jülich, 52425 Jülich, Germany; 7 Laboratory of Biomolecular NMR, St. Petersburg State University, St. Petersburg 199034, Russia

## Abstract

The large majority of three-dimensional structures of biological macromolecules have been determined by X-ray diffraction of crystalline samples. High-resolution structure determination crucially depends on the homogeneity of the protein crystal. Overall ‘rocking' motion of molecules in the crystal is expected to influence diffraction quality, and such motion may therefore affect the process of solving crystal structures. Yet, so far overall molecular motion has not directly been observed in protein crystals, and the timescale of such dynamics remains unclear. Here we use solid-state NMR, X-ray diffraction methods and μs-long molecular dynamics simulations to directly characterize the rigid-body motion of a protein in different crystal forms. For ubiquitin crystals investigated in this study we determine the range of possible correlation times of rocking motion, 0.1–100 μs. The amplitude of rocking varies from one crystal form to another and is correlated with the resolution obtainable in X-ray diffraction experiments.

## Abstract (web summary)

Small-amplitude overall motion of molecules in crystals limits the achievable resolution in X-ray diffraction, yet little is known about its exact nature. Here, the authors obtain NMR, XRD and MD data from three different crystal forms of a protein (ubiquitin) to gain insight into amplitude and timescale of such motions.

## Graphical abstract

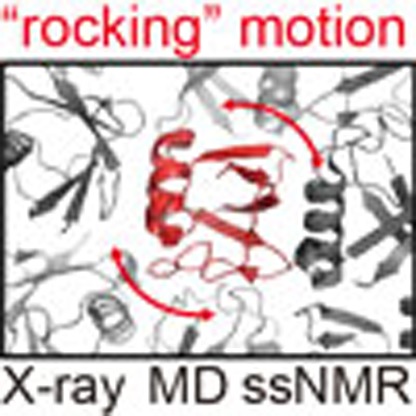

X-ray crystallography is the quintessential method for macromolecular structure determination. The method provides atomic coordinates along with atomic displacement parameters, which are generally expressed as B-factors and reflect the coordinate uncertainty around the mean positions. The coordinate precision in X-ray structures is limited by several factors, including model errors and invalid restraints^1^. The precision is also adversely affected by protein dynamics and static disorder, which together contribute to the ‘blurring' of electron density maps. Motion has therefore long been treated as a nuisance limiting the effective resolution at which a crystallographic structure can be solved. Recent methodological advances have shown, however, that useful dynamical information can be extracted from X-ray diffraction (XRD) data^2^,^3^,^4^,^5^,^6^,^7^,^8^,^9^,^10^, provided that high-resolution structural information is available. Several investigators pointed out the importance of rigid-body motions, which limit the achievable resolution in XRD experiments^4^,^5^,^6^,^7^,^8^,^9^.

Overall motion is routinely modelled from XRD data using translation-libration-screw (TLS) analyses. However, refined TLS parameters offer only a simplified view of rotational and translational dynamics in the crystal lattice, meaning that some ambiguity remains regarding the physical nature of the modelled motion. Furthermore, diffraction data cannot provide insights into the timescale of motions, making it difficult to distinguish between static disorder and molecular motions. In other words, it is not possible to ascertain that the dynamics modeled from XRD data accurately reflect the overall motion of the molecules in the crystal.

Magic-angle spinning (MAS) NMR spectroscopy provides atomic-level-resolution access to crystalline proteins. MAS NMR is complementary to XRD in the sense that it can provide atom-specific insights into reorientational motions at a large number of sites. A number of NMR observables, in particular relaxation rate constants and dipolar couplings, probe exclusively the angular motion as sensed at each individual site while being unaffected by static disorder. Furthermore, NMR measurements can provide direct access to the timescale at which dynamics occur. It has been hypothesized before that rocking motion in crystals might be observable through spin relaxation parameters in MAS NMR^11^, yet no experimental evidence has to date been produced. Rotational diffusion and its effects have been investigated for membrane proteins embedded in lipid bilayers^12^,^13^,^14^,^15^, but reorientational fluctuations in protein crystals remain largely unexplored.

Here we report on the combined use of MAS NMR, XRD and microsecond-long molecular dynamics (MD) simulations of explicit crystal lattices to characterize the overall rocking motion and the local internal dynamics of the protein ubiquitin in three different crystal forms. Our results provide direct insight into the amplitudes and timescales of rocking motion in the three crystals. They illuminate the possibly general relationship that exists between crystalline rocking motions and the experimental resolution achieved in XRD and MAS NMR experiments.

## Results

### MAS NMR and XRD of three different ubiquitin crystals

Disentangling overall rigid-body motion (herein referred to as ‘rocking' motion) from internal dynamics is a challenge, regardless of whether XRD or MAS NMR is used as an experimental tool. This is because both types of motion contribute to the dynamics-related observables, that is, to B-factors in XRD and to relaxation and dipolar-coupling parameters in MAS NMR. In the present study, these complications were circumvented by using different crystal forms of the same protein, allowing us to assume that the internal dynamics are similar—an assumption that we verify below—and thus to focus on differences in overall motion of the protein in the crystal lattices.

We prepared three different crystal forms of the 8-kDa globular protein ubiquitin. These crystals are henceforth referred to as MPD-ub, cubic-PEG-ub and rod-PEG-ub, reflecting the different precipitation agents (methyl-pentanediol (MPD) and polyethylene glycol (PEG), respectively) and the morphology of the crystals. Structures for the three crystal forms have been solved before and correspond to Protein Data Bank entries 3ONS (ref. 16), 3N30 (ref. 17) and 3EHV (ref. 18), respectively. To ensure that our crystals were consistent with the previously reported structures, XRD data were collected on the three crystals. For the two types of PEG crystals, we collected diffraction data at 100 K and solved the structures by molecular replacement, confirming the identity to the two already reported sets of coordinates. Our MPD-ub crystals appeared too thin for conventional structure determination when crystallized under the conditions that yield high-quality MAS NMR spectra. Nevertheless, a powder pattern obtained by rotating a scoop of MPD-ub crystals into the X-ray beam yielded a distribution of Bragg peaks similar to that calculated from the previously deposited structure (see Methods section). Thus, our crystals display the same space group as crystals previously obtained in the same crystallization conditions.

**Figure 1:**
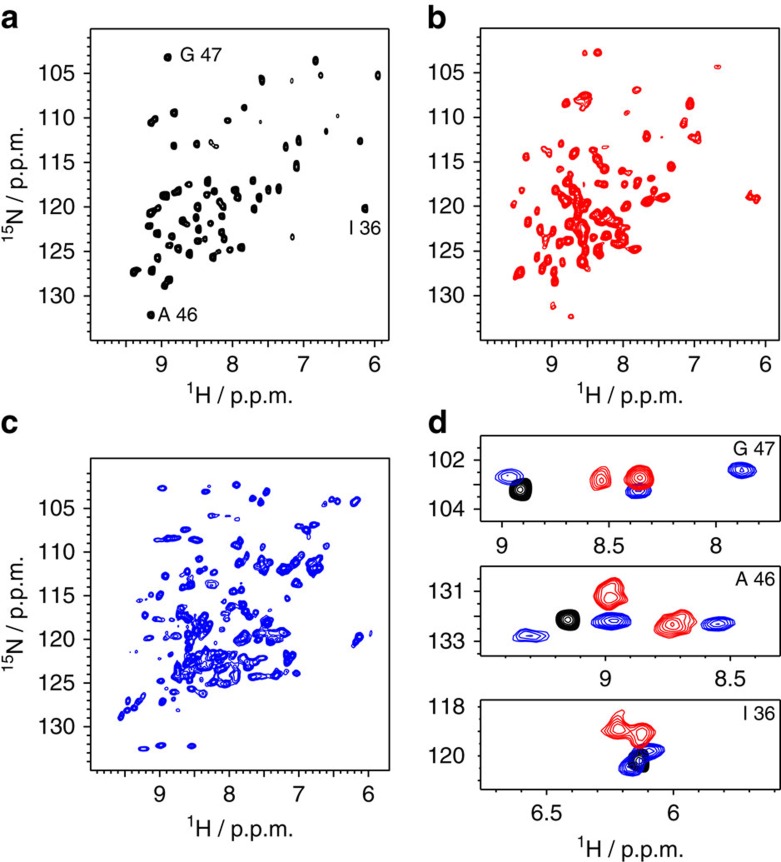
High-resolution solid-state NMR spectra of three different crystal forms of ubiquitin. ^1^H–^15^N NMR spectra of MPD-ub, cubic-PEG-ub and rod-PEG-ub are shown in **a**–**c**, respectively. (**d**) Three regions of the spectra with well-isolated peaks, showing the different peak multiplicity observed in the different crystals (the residue numbers are indicated in each subpanel). A set of assigned HN and NCA spectra as well as methyl H-C spectra are shown as Supplementary Figs 1, 2 and 3, respectively.

We used MAS NMR to further study the three crystal forms and obtain information about their dynamics. Figure 1 shows MAS NMR ^1^H–^15^N correlation spectra recorded on the three crystal forms. A first interesting observation concerns the number of peaks found in the three spectra. In MPD-ub, which has been extensively characterized before^19^,^20^,^21^, one set of well-resolved ^1^H–^15^N cross-peaks is observed. In cubic-PEG-ub many residues give rise to two peaks, as exemplified in Fig. 1d. In rod-PEG-ub we find—for several instances of well-isolated regions of the spectrum—three peaks per residue. This peak multiplicity is in good agreement with the number of non-equivalent molecules in the asymmetric unit of the crystals, i.e. one (MPD-ub), two (cubic-PEG-ub) and three (rod-PEG-ub), respectively. Of note, similar peak duplication has been reported previously in NMR spectra of ubiquitin crystals (prepared under slightly different conditions and resulting in different NMR spectra) and polymorphs of GB1 crystals^22^,^23^,^24^,^25^. We obtained residue-specific assignments of a majority of HN resonances in cubic-PEG-ub, using a set of ^1^H- and ^13^C-detected three-dimensional correlation spectra (assignments are reported in Supplementary Table 1). Owing to the higher spectral complexity arising from the three non-equivalent molecules, we did not assign the spectra of rod-PEG-ub.

### Internal dynamics in different crystals from MAS NMR and MD

We conducted ^1^H^N^-detected ssNMR experiments on highly deuterated protein samples to study dynamics in MPD-ub and cubic-PEG-ub. In what follows, we rely on three different experimental observables that concurrently probe a wide range of timescales at each amide site in the protein and are informative of both amplitudes and timescales of the dynamics. The first parameter, ^1^H–^15^N dipolar-coupling derived squared order parameter *S*^2^, report on the amplitude of motion of HN bond vectors. The value of *S*^2^ can range from 1 for a completely rigid bond to 0 for fully dynamically disordered peptide planes. The dipolar-coupling derived order parameters reflect the net effect from all reorientational motions occurring on timescales shorter than about 100 μs. The second parameter, the ^15^N *R*_1_ spin relaxation rate constant, is sensitive to both the amplitude and the timescale of ^1^H–^15^N bond vector motions. This relaxation parameter is particularly sensitive to dynamics on timescales from tens of picoseconds to ∼100 nanoseconds (Supplementary Fig. 4). The third parameter, the ^15^N *R*_1*ρ*_ spin relaxation rate constant, is also sensitive to both the amplitude and timescale of the motion, but mainly to slower motion, occurring on the ns–μs timescale (see Supplementary Fig. 5 and discussion below). Analysing these three experimental observables therefore provides good insight into motional properties of individual protein residues over a wide range of timescales.

**Figure 2:**
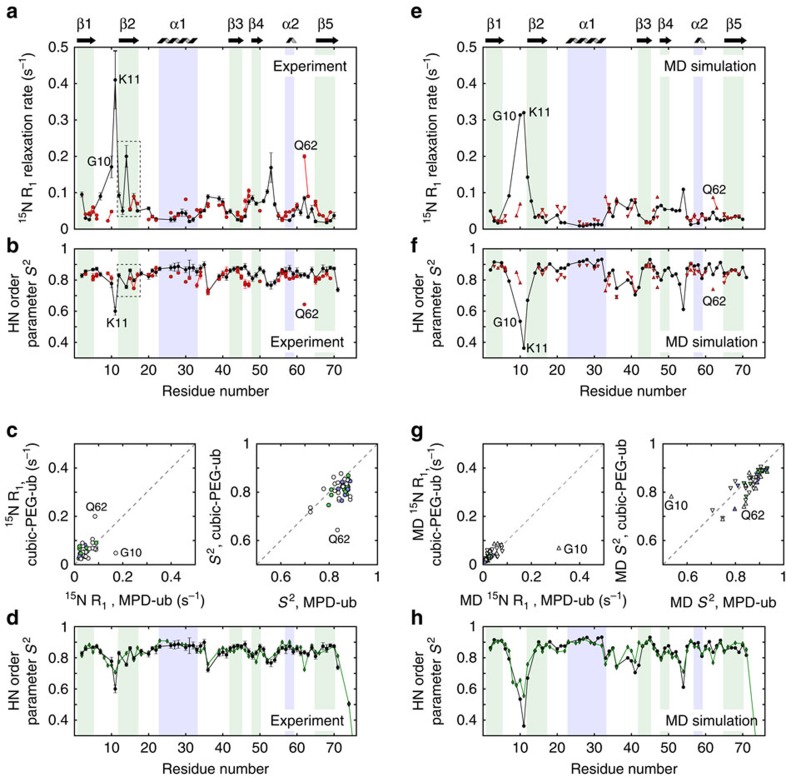
Site-resolved HN dynamics parameters in two different crystal forms from NMR experiments and MD simulations. Per-residue dynamics data obtained from MPD-ub (black), cubic-PEG-ub (red) and ubiquitin in solution (green) as observed by NMR experiments (**a**–**d**) and MD simulations (**e**–**h**). (**a**) Experimental ^15^N *R*_1_ rate constants and (**b**) dipolar–coupling derived squared order parameters, *S*^2^. In cases where two data points per residue could be obtained in cubic-PEG-ub, corresponding to the pair of non-equivalent molecules, these are represented by two distinct symbols. Because of the spectral overlaps in spectra of cubic-PEG-ub, it was not possible to unambiguously assign all signals to chain A or B; those data points that have been identified as belonging to the same chain are connected by a solid line. Secondary-structure regions are indicated by the shaded bands and identified above the plot. (**c**) Correlations between the data from two different crystal forms; symbols are coloured according to the secondary-structure classification (α-helix in blue and β-strands in light green). (**d**) Experimental *S*^2^ values measured in MPD-ub crystals (black) juxtaposed on *S*^2^ values from solution-state measurements (green, ref. 57). Supplementary Table 2 lists experimental data for cubic-PEG-ub. Data for MPD-ub have been reported elsewhere^20^,^26^. Data in **e**–**h** are from MD simulations, plotted using the same template and colouring conventions as in the case of the experimental data (**a**–**d**). The data points from chains A and B in cubic-PEG-ub simulation are plotted with downward- and upward-pointing red triangles, respectively. Supplementary Tables 3–5 list the simulated parameters for MPD-ub, cubic-PEG-ub and ubiquitin in solution, respectively.

**Figure 3:**
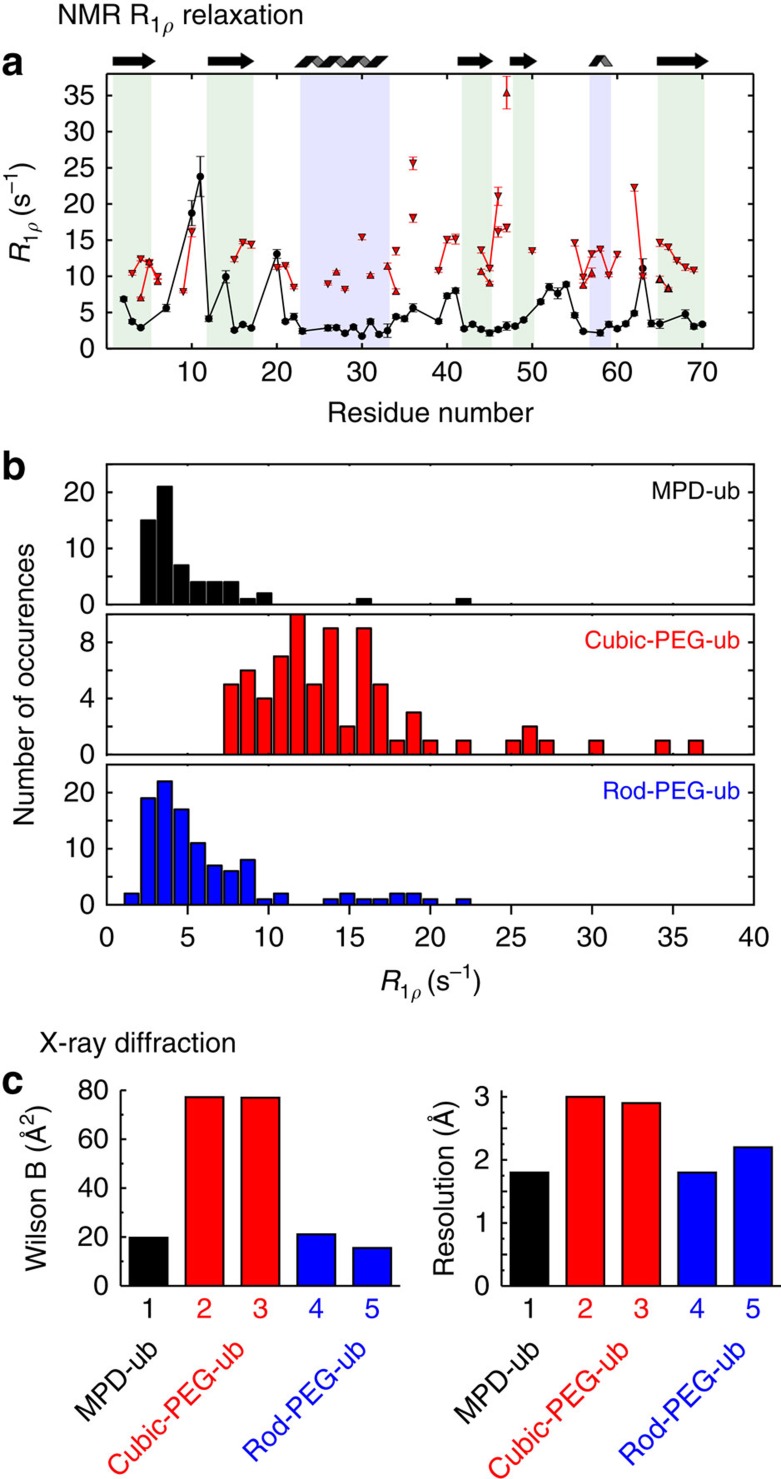
Evidence for rigid-body motion (rocking) in ubiquitin crystals from NMR and XRD data. (**a**) Residue-wise ^15^N *R*_1*ρ*_ spin relaxation rate constants in MPD-ub (black) and cubic-PEG-ub (red). (**b**) Histograms of per-residue ^15^N *R*_1*ρ*_ relaxation rate constants in the above two crystals, as well as rod-PEG-ub (blue). (**c**) XRD data pointing to different motional behaviour of ubiquitin in the three crystals: Wilson B-factors (left) and structural resolution (right). Shown are the data from the following five PDB structures: 1, 3ONS (ref. 16); 2, 3N30 (ref. 17); 3, 4XOL (this study); 4, 3EHV (ref. 18); 5, 4XOK (this study).

Figure 2a–d shows a comparison of site-specific amide ^15^N *R*_1_ rate constants and NH order parameters in MPD-ub and cubic-PEG-ub, obtained at 300 K sample temperature. These data reveal that the local dynamics in the two crystal forms are generally similar, with few differences. Overall, residues located in secondary structure elements have high order parameters *S*^2^ and low *R*_1_ relaxation rate constants, indicating that these residues are motionally restricted in both crystal forms. Previous studies of MPD-ub showed that low-amplitude motions in the secondary-structure elements occur primarily on the picosecond timescale^20^. Certain details of local dynamics are reproduced in both crystals. For example, an alternating pattern of low/high motional amplitudes in strand β2 is observed in both MPD-ub and cubic-PEG-ub (residues T12–V17, dashed outline in Fig. 2). This pattern arises from alternation of amides which are hydrogen bonded or otherwise exposed to solvent^26^. Similarities between the two crystals are also found in several loop regions, such as the α1–β3 loop and the β3–β4 loop, which show similarly increased flexibility (as reflected in the increased *R*_1_ and decreased *S*^2^ values). Yet, distinct differences in dynamic behaviour are observed at certain sites, as evident from Fig. 2a,b. For example, high *R*_1_, low *S*^2^ and high *R*_1*ρ*_ (see further below, Fig. 3) values in the β1–β2 loop in MPD-ub are indicative of extensive ns-timescale motion. In contrast, this loop appears rigid in cubic-PEG-ub, displaying similar dynamics to residues in the secondary-structure regions. Another prominent example is residue Q62 located in the α2–β5 loop, which displays significant flexibility in cubic-PEG-ub but seems relatively stiff in MPD-ub. It is also worth noting that the order parameters in MPD-ub are overall slightly higher than in cubic-PEG-ub. When applying an overall scaling factor of 1.04 to the *S*^2^ values from cubic-PEG-ub, the agreement with MPD-ub data is significantly improved (see Supplementary Fig. 6 for details). As discussed further below, this offset can be explained by the rocking motion of ubiquitin within the crystal lattice of cubic-PEG-ub.

It has been recently shown that experimental data by MAS NMR and XRD can be successfully reproduced using explicit MD models of protein crystals^27^,^28^,^29^. Towards this goal we have recorded 1-μs-long all-atom MD trajectories representing the two different crystal lattice arrangements of ubiquitin. A block of four crystal unit cells (24 ubiquitin molecules) was simulated for MPD-ub, while one crystal unit cell (48 ubiquitin molecules) was simulated for cubic-PEG-ub. The presence of multiple protein molecules in the simulations effectively improves the statistical properties of the MD models. The results from MD simulations, Fig. 2e–h, nicely reproduce the experimentally observed trends. Consistent with the experimental data, simulated ^15^N *R*_1_ and *S*^2^ parameters are overall similar in the two crystals, with two notable exceptions found in the β1–β2 loop and residue Q62. On average, the simulated *S*^2^ in cubic-PEG-ub are slightly lower than those in MPD-ub, which is again consistent with the experimental observations.

For the two crystal forms at hand, NMR and MD produce similar *R*_1_ profiles (sensitive primarily to motions on a timescale of tens of picoseconds to ∼100 nanoseconds) and *S*^2^ profiles (sensitive to all motions faster than ca. 100 μs). This leads us to suggest that internal dynamics of ubiquitin are similar in the two crystals. Furthermore, site-specific *S*^2^ data in crystals are remarkably similar to those in solution, as confirmed by experimental measurements as well as MD simulations (Fig. 2d,h). These observations are in line with the results from previous studies, which suggested that the crystalline environment has only comparatively minor effect on protein internal dynamics^30^,^31^,^32^,^33^,^34^,^35^,^36^,^37^.

### Evidence for overall rocking motion from MAS NMR and MD

Having established that internal motions on ps–ns timescales are generally similar in the two crystals, we then focused on amide-^15^N *R*_1*ρ*_ spin relaxation rate constants. This relaxation parameter is highly sensitive to amplitudes and time constants of reorientational motions occurring on longer timescales—specifically nanosecond to microsecond motions (Supplementary Fig. 5). The experimental *R*_1*ρ*_ relaxation rate constants in MPD-ub and cubic-PEG-ub are summarized in Fig. 3a. Interestingly, a clear-cut difference is observed between the two crystal forms. In particular, the ‘base' level of *R*_1ρ_ within secondary structure regions is significantly higher in cubic-PEG-ub (12 s^−1^) than in MPD-ub (3.5 s^−1^). To a reasonable approximation this offset is uniform across the sequence, at least for secondary-structure elements. Site-specific differences in *R*_1*ρ*_ rates are found mostly in loops, and can be ascribed to nanosecond mobility of these regions^20^,^26^; differences in loop dynamics have been exposed already by the *R*_1_ and order parameter data discussed above.

The overall offset in the ‘base' *R*_1*ρ*_ rates of the two crystals points to a global motion that involves the entire molecule. This motion appears to be present in cubic-PEG-ub crystals, but absent or less pronounced in MPD-ub crystals. We attribute this effect to relatively slow reorientational fluctuations of the protein molecule embedded in the crystal lattice, that is, to rocking motion. In what follows, we will show that the observed *R*_1*ρ*_ offset in cubic-PEG-ub is consistent with a rocking motion having an amplitude of several degrees and a correlation time in the range from hundreds of nanoseconds to tens of microseconds.

**Figure 4:**
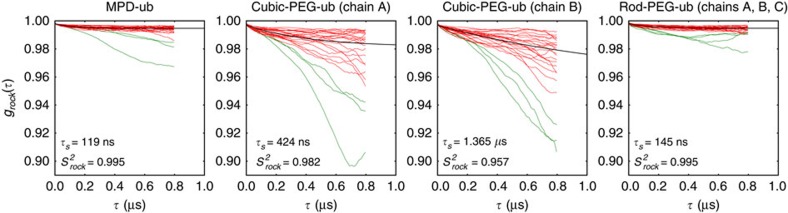
Rocking correlation functions from three 1-μs-long MD trajectories of ubiquitin crystals. The curves, representing individual ubiquitin molecules in the crystals, were averaged and then fitted using a bi-exponential function with a flat base, 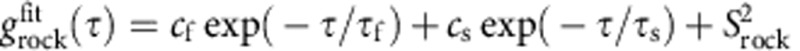. The best-fit curve 
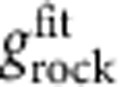
 (*τ*) is shown in the plot (black line), along with the values of the fitted parameters *τ*_s_ and 
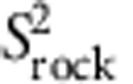
. In the case of cubic-PEG-ub we have treated two inequivalent molecules, chains A and B, separately, whereas in the case of rod-PEG-ub the data from three inequivalent molecules, chains A, B and C, have been averaged before the fitting. Only red curves have been used in the fitting procedure (green curves have been classified as outliers and set aside).

To obtain additional insight into rocking motion, we analysed the 1-μs-long MD trajectories of the three crystals (MPD-ub and cubic-PEG-ub, as described previously, as well as rod-PEG-ub). For each trajectory we defined a set of reference coordinates, that is, a block of crystal unit cells constructed from the corresponding crystallographic structures. We further calculated rotation matrices Ξ connecting instantaneous MD coordinates of protein molecules with their respective reference coordinates (Ξ were obtained from least-square fitting of the Cα atoms belonging to the protein secondary structure). A sequence of these small-angle rotation matrices encodes the rocking motion of each individual ubiquitin molecule. Finally, matrices Ξ have been applied to a set of 100 dipolar vectors uniformly distributed on a unit sphere so as to calculate ‘isotropic' rocking correlation functions *g*_rock_(*τ*). The results are shown in Fig. 4 for all individual ubiquitin molecules from MPD-ub, cubic-PEG-ub and rod-PEG-ub simulations. Supplementary Movies 1–3 illustrate rocking motion in MPD-ub, cubic-PEG-ub (chain A) and cubic-PEG-ub (chain B), respectively.

Clearly, the rocking motion found in the MD simulation of cubic-PEG-ub (order parameters 0.982 and 0.957 for chains A and B, respectively) is much more pronounced than for MPD-ub and rod-PEG-ub (average order parameter 0.995 for both systems). This result correlates well with our experimental data that offer multiple lines of evidence for increased rocking motion in cubic-PEG-ub. The MD simulations also have a potential to shed light on the timescale of rocking dynamics. The simulated correlation functions *g*_rock_(*τ*) shown in Fig. 4 involve a small-amplitude fast component with the correlation time *τ*_f_∼1 ns and the more prominent slow component with *τ*_s_ in the range from ∼0.1 to 1 μs.

It is important to bear in mind, however, that MD simulations offer, at best, a qualitative insight into rocking motions. The effect of crystal packing in protein crystals is governed by a multitude of subtle interactions that involve, in particular, mobile side chains and hydration water. Capturing these interactions in the context of MD modelling remains a challenge even for state-of-the-art force fields. As a consequence, the crystal lattice undergoes slight but progressive distortion during the course of the simulation^38^. Of note, such ‘structural drift' has also been observed in MD simulations of globular proteins, even though the determinants of protein structure (for example, amide hydrogen bonds) are generally far better understood than the determinants of crystal packing^39^. This leads to a situation where rocking motion in the MD simulations occurs against the background of gradually deteriorating crystal lattice.

One should also be aware of statistical limitations. Even though each of our 1-μs-long trajectories contains from 24 to 48 ubiquitin molecules, which improves their statistical properties, this would not be sufficient to capture rocking dynamics should it occur on a timescale approaching 100 μs. Note that in this situation it can be difficult to differentiate between ‘structural drift' (discussed above) and lack of convergence. The limitations of the MD model can be appreciated from Fig. 4 where one observes a significant spread in the rocking correlation functions belonging to the individual ubiquitin molecules, including a number of outliers (green curves). Under these circumstances it is impossible to meaningfully estimate the anisotropy of rocking motion, although in general rocking is certainly expected to be anisotropic. For further insight into convergence properties of *g*_rock_(*τ*) see Supplementary Fig. 7.

Finally, one should bear in mind that no attempt has been made to include into MD simulations the crystallization additives, such as 2-methyl-2,4-pentanediol or PEG. These compounds do not appear in the crystallographic coordinates and it is unclear to what degree they are partitioned into the crystals. We also did not include the Zn^2+^ ions, although they are explicitly present in the X-ray structures of cubic-PEG-ub and rod-PEG-ub. There are currently no force field parameters that would be suitable to model Zn^2+^ ions in highly diverse and conformationally dynamic adventitious binding sites at protein–protein interfaces. Fundamentally, no single set of force-field parameters would be sufficient in this situation^40^,^41^,^42^.

**Figure 5:**
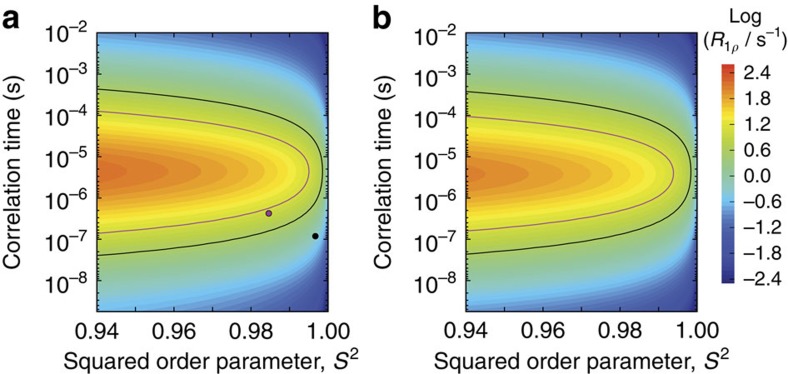
Estimating the timescale of rocking motion from ^15^N *R*_1*ρ*_ measurements. Plotted is the ^15^N *R*_1*ρ*_ relaxation rate constant as a function of the order parameter *S*^2^ and correlation time *τ* that describe the motion of the NH vector. (**a**) The calculations were conducted using the Redfield-theory formulas, equations 8 and 18 in ref. 65. (**b**) Alternatively, the calculations were conducted using a numeric model that is also valid outside the Redfield regime; the geometrical details of this two-site jump model are exactly as described in Fig. 2 of ref. 66, and the simulation was implemented in the program GAMMA^67^, as described before^68^. The jump angle Φ used in the numerical simulation is related to the order parameter according to *S*^2^=(1+3 cos^2^ Φ)/4. Both calculations **a** and **b** assume an MAS frequency of 39.5 kHz and a ^15^N spin-lock radio-frequency field strength of 15 kHz, the same as in our experimental measurements. The results obtained from the two computational models prove to be similar, thus validating the Redfield-theory based approach for the problem at hand (see Supplemetary Fig. 5 for additional discussion). The black contour line represents the ‘base' *R*_1*ρ*_ relaxation rate constant as experimentally found in MPD-ub (3.5 s^−1^), whereas the purple line represents the ‘base' rate in cubic-PEG-ub (12 s^−1^). The black circle represents the relaxation due to rocking motion as obtained from the MD trajectory of MPD-ub, while the purple circle represents the relaxation due to rocking motion in cubic-PEG-ub (chain A). These relaxation rate constants were calculated based on the respective correlation functions 
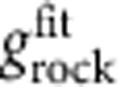
 (*τ*), see Fig. 4. In doing so, the small rapidly decaying component of the correlation function, *τ*_f_∼1 ns, has been ignored since it makes only negligible contribution to *R*_1*ρ*_. Thus, for the purpose of calculating *R*_1*ρ*_ we have made the identification 1−*S*^2^=*c*_s_ and *τ*=*τ*_s_, where *c*_s_ is the amplitude of the slow rocking motion and *τ*_s_ is the respective time constant. Note that the experimentally determined relaxation rate constants (black and purple contour lines) reflect both rocking motions and internal protein dynamics, whereas the calculated rates (black and purple circles) are limited to rocking alone.

Nevertheless, despite all these shortcomings, our MD simulations clearly reproduce the same trend as has been observed experimentally and thus confirm that MPD-ub and rod-PEG-ub form stable crystal arrangements, whereas cubic-PEG-ub is prone to rocking. Furthermore, the MD-derived correlation functions 
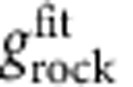
 (*τ*) can be used to calculate the contributions of rocking motion into *R*_1*ρ*_ relaxation rate constants. These contributions turn out to be 0.6 s^−1^ for MPD-ub, 9.1 and 63.4 s^−1^ for cubic-PEG-ub (chains A and B, respectively) and 0.7 s^−1^ for rod-PEG-ub. The difference between the first two numbers, 8.5 s^−1^, reproduces quantitatively the difference between the experimentally measured *R*_1*ρ*_ rates in MPD-ub (base rate 3.5 s^−1^) and cubic-PEG-ub (base rate 12 s^−1^). Although this result is certainly fortuitous, it demonstrates the potential for quantitative analysis of rocking dynamics using MD models (see Fig. 5 for further details).

In order to obtain better insight into the time scale of the rocking motion, we plot in Fig. 5 the calculated R_1ρ_ relaxation rate constant as a function of the amplitude and time scale of the motion. The black curve shows the solutions (order parameters and correlation times) that are in agreement with the experimentally measured 'base' *R*_1*ρ*_ rate in MPD-ub, while the purple curve shows the solutions for cubic-PEG-ub. Furthermore, the black and purple circles illustrate the results obtained from the two respective MD trajectories. If one takes guidance from the MD trajectory of cubic-PEG-ub, and specifically the results for chain A (purple circle in the plot), then one is led to believe that rocking motion is characterized by *S*^2^∼0.985, *τ*_s_∼400 ns. Indeed, such a scenario would be consistent with all of our existing experimental data (Fig. 5). However, as explained above, the MD simulations offer only qualitative insight into the problem and cannot be viewed in this case as a source of quantitative information. Therefore, we recognize that there is an alternative solution corresponding to the upper branch of the purple curve in Fig. 5: *S*^2^∼0.985, *τ*_s_∼40 μs. Generally, we can safely conclude that rocking motion in cubic-PEG-ub occurs on the timescale from hundreds of nanoseconds to tens of microseconds. More accurate determination of this important parameter is deferred to future work.

The emerging picture is self-consistent in more ways than one. For instance, MD simulations predict that order parameters in the cubic-PEG-ub crystal should be ∼2–3% lower than in MPD-ub due to the intensified rocking motion. This is compatible with our experimental data, which show that cubic-PEG-ub order parameters *S*^2^ are ∼4% lower than those in MPD-ub (see above and Supplementary Fig. 6). Furthermore, the MD model predicts the crystallographic B-factors in cubic-PEG-ub to be significantly higher than in MPD-ub, with rocking motion making an important contribution to B-factors in cubic-PEG-ub, but much less in MPD-ub (Supplementary Fig. 8). These predictions are also borne out by the experimental data, as explained below.

### Overall rocking impacts resolution in XRD experiments

Both the NMR and MD data indicate that ubiquitin molecules arranged in a crystal lattice experience varying degree of rocking motion at room temperature. But is this rocking motion impacting the XRD data collected at 100 K? Figure 3c shows that this is indeed the case. The Wilson B-factor in cubic-PEG-ub is almost fourfold higher than in MPD-ub and the resolution is significantly lower, which we propose to arise from differences in the respective rocking dynamics. This correlation between NMR ^15^N *R*_1*ρ*_ relaxation data and XRD resolution is further substantiated by the third crystal form, rod-PEG-ub, which displays lower ^15^N *R*_1*ρ*_ rates, suggesting that rocking motions are of low amplitude (blue bars in Fig. 3b). Correspondingly, these rod-PEG-ub crystals display a lower Wilson B, and they diffract to high resolution (blue bars in Fig. 3c).

Similar conclusions can also be reached if a TLS model is used to account for rigid-body motion of proteins in the crystals^9^. In XRD refinement, TLS modelling is one of the ways by which collective and local motions can be separated. As expected, cubic-PEG-ub shows the highest librational as well as translational amplitude among the three crystal structures (Supplementary Fig. 9), in good qualitative agreement with our NMR and MD data. At this stage, it should be reminded that the TLS model is based on certain simplifying assumptions. If a protein molecule experiences a series of small rotations with different pivot points (a likely scenario in the protein crystal lattice), the TLS model may interpret this dynamics as translation. In this sense, the information content of the TLS parameters is not very different from that of the Wilson B-factor insofar as it is difficult to disentangle libration and translation.

It is interesting to examine why the same molecule, with overall identical structure and internal dynamics, exhibits more rocking motion in one of the examined crystals than in others. A direct influence on rocking of the precipitating agent used for crystallization can be excluded on the basis that both cubic-PEG-ub and rod-PEG-ub crystals crystallize in essentially the same condition (sometimes even in the same crystallization drop). The amplitude of the rocking motion is likely to be influenced by the crystal packing density—increased contact surface area is generally expected to offer more resistance to rocking. In our case, the packing density is indeed lowest for the crystal with the most pronounced rocking motion, with solvent content *V*_s_ of 58% for cubic-PEG-ub, 49% for MPD-ub and 40% for rod-PEG-ub, respectively. These values follow the expected trend—lower packing density allows for more overall motion. However, given the small size of this data set, the correspondence of rocking motion and packing density may as well be fortuitous. We thus performed a wider analysis seeking to determine whether there is a correlation between packing density and rocking dynamics (as manifested in XRD resolution and B-factors). A comprehensive search of the Protein Data Bank indeed shows that high solvent content correlates with low resolution and high Wilson B, with correlation coefficients of 0.39 and 0.36, respectively (Supplementary Fig. 10a). As expected, these dependencies are subject to strong scatter, reflecting the intricate and complex nature of the crystallization process and the large diversity of the shapes and properties of the analysed structures^43^,^44^. We have also repeated this analysis for the subset of crystallographic structures in the Protein Data Bank that have been solved at room temperature. The results prove to be very similar (cf. Supplementary Fig. 10a,b). Although not a direct proof, this finding suggests that the spread of orientations observed at cryo-temperatures (typically 100 K) reflects qualitatively the amplitudes of rocking motions at room temperature. In other words, the disorder associated with rocking motion also persists under cryo-cooling conditions.

## Discussion

We have shown here that three independent and complementary techniques, NMR, MD and XRD, all provide evidence for an overall rocking motion in protein crystals. The rocking motion is (i) observed by NMR, through the increased *R*_1*ρ*_ rates, as well as a slight decrease of order parameters; (ii) reproduced by MD in all-atom crystal lattice simulations; and (iii) confirmed by XRD through the decreased resolution and increased atomic displacement factors. We have been able to provide for the first time a measure of the timescale at which this motion takes place at room temperature, which turned out to be hundreds of nanoseconds to tens of microseconds. Our data suggest that rigid-body motion is an important determinant for the resolution achieved in X-ray crystallography and may explain at least partly why visually perfect crystals do not always produce high-resolution XRD data^45^.

## Methods

### Sample preparation

Uniformly [^2^H,^13^C,^15^N]-labelled ubiquitin was obtained by bacterial overexpression in *Escherichia coli* and purified using ion-exchange and size-exclusion chromatography. The protein was dialysed against water, lyophilized and then resuspended in 20 mM ammonium acetate at pH 4.3 with protein concentration of 20 mg ml^−1^. All crystals were obtained using a sitting-drop crystallization plate with 47–50 μl protein drops and 500 μl reservoir buffer. In all protein drops except MPD-ub, the protein solution was mixed with reservoir buffer at a ratio of 1:1. All NMR samples have been prepared with H_2_O:D_2_O ratio of 1:1 (taking into account the exchangeable protons on precipitation agents).

For generating MPD-ub crystals, described before^19^, the ubiquitin solution was mixed with reservoir buffer at a ratio of 3.7:1. The reservoir buffer was a mixture of 20 mM citric acid, pH 4.2 and 2-methyl-2,4-pentanediol (MPD) at a ratio of 40:60. Needle-shaped crystals were obtained at 4 °C after about 1–2 weeks.

Cubic-PEG-ub crystals (PDB ID code 4XOL) were obtained with a reservoir buffer of 100 mM 2-(N-morpholino)ethanesulfonic acid (MES), pH 6.3, 20% PEG 3350 and 100 mM zinc acetate. Cubic-shape crystals were obtained within 1 week at 23 °C.

Rod-PEG-ub crystals (PDB ID code 4XOK) were obtained with a reservoir buffer of 50 mM 4-(2-hydroxyethyl)-1-piperazineethanesulfonic acid (HEPES), pH 7.0, 25% PEG 1500 and 25 mM zinc acetate. Long-rod-shape crystals were obtained after 2 weeks at 23 °C.

In addition to these three crystal forms, we also obtained a fourth crystal, from unlabelled ubiquitin. This crystal, rod-PEG-ub-II, (PDB ID code 4XOF) was obtained with a reservoir buffer of 50 mM MES, pH 6.3, 25% PEG 2000 and 1 mM zinc acetate, after 1 month at 23 °C. The amount of crystals obtained was insufficient for NMR analyses, but we were able to determine its structure by XRD.

For the preparation of NMR samples, protein crystals with their crystallization solution were pipetted into an in-house made centrifugation device (funnel) that was adapted to a 1.6-mm solid-state NMR rotor. The device, similar to a recently reported filling tool^46^, was spun in a Beckman SW41 rotor at 10,000 r.p.m. (about 15,000*g*) for 10 min to pellet the protein crystals into the NMR rotor. Typical samples contained ∼4–5 mg of material (total mass, including the solvent).

### NMR spectroscopy

All dynamics experiments were performed on an Agilent VNMRS spectrometer operating at a ^1^H Larmor frequency of 600 MHz, equipped with a 1.6 mm HXY MAS probe tuned to ^1^H, ^13^C and ^15^N frequencies. HN dipolar couplings as well as ^15^N *R*_1_ and ^15^N *R*_1*ρ*_ relaxation rate constants were measured using proton-detected two-dimensional HN correlation experiments, identical to those used before, employing MAS frequencies between 37.0 (dipolar-coupling measurement) and 39.5 kHz (*R*_1*ρ*_ measurement, using a ^15^N spin-lock with radio-frequency field strength of 15 kHz)^20^. The REDOR scheme^47^ was used to measure HN dipolar couplings; this experiment was shown to be particularly robust with respect to systematic errors^48^. Dipolar couplings were fitted based on peak volumes in a series of two-dimensional HN spectra with variable recoupling time. The employed χ^2^ fitting procedure explicitly takes into consideration the radio-frequency field inhomogeneity across the sample as described^20^ and utilizes full-scale numerical simulations of the REDOR recoupling element conducted on a grid which samples different coupling strengths. Error margins were obtained from Monte Carlo analyses, based on three times the spectral noise level. Relaxation rate constants were obtained through numerical fits using a single-exponential function and their associated error margins were also obtained from Monte Carlo analysis.

Resonance assignment of MPD-ub has been reported before^19^,^26^. Assignment of cubic-PEG-ub has been achieved using a series of three-dimensional correlation spectra based on ^13^C detection (NCACX with 50 ms DARR CC transfer, NCOCX with 50 ms DARR CC transfer and CANCO, NCACB with DREAM transfer) and spectra with ^1^H detection (hCONH, hCANH, hcoCAcoNH)^49^. For a number of residues two sets of spectral correlations were identified, resulting from the two non-equivalent molecules in the unit cell (chains A and B). It was possible to obtain partial connectivities for certain groups of peaks representing chain A or, alternatively, chain B. It was not possible to unambiguously identify the two sets of resonances, because of the extensive chemical shift overlap between the two sub-spectra. The obtained partial connectivities are shown by red lines in Figs 2 and 3.

### MD simulations and analysis

The initial coordinates for the MPD-ub simulation were obtained from the crystallographic structure 3ONS (ref. 16). Four flexible C-terminal residues of ubiquitin were rebuilt as described previously^28^. To determine the protonation status of ionizable residues, we performed the PROPKA^50^ calculations for ubiquitin in the relevant crystal-lattice environment. The effective pH was assumed to be 4.2, same as in the crystallization buffer of 3ONS. The original dimensions of the unit crystal cell were all multiplied by a factor 1.016 to account for thermal expansion of the protein crystal on transition from 100 (temperature at which 3ONS was solved) to 301 K^51^. The unit crystal cell was hydrated using SPC/E water^52^; in doing so, the crystallographic water molecules have been retained in their original positions. The system was neutralized by adding Cl^−^ ions. The periodic boundary box was defined as a block of four crystal unit cells, containing 24 ubiquitin molecules and 8,772 water molecules, for the total of 56,244 atoms. The simulations were conducted under Amber ff99SB*-ILDN force field using Amber 11 program^53^,^54^,^55^. The trajectory was recorded at 301 K, using isothermal-isobaric (NPT) ensemble. The volume of the simulation box remains stable throughout the simulation within 0.5% of its target value (on average, there is a slight uniform expansion as described by linear factor 1.0009). The production rate with NVIDIA GeForce GTX580 cards was 9 ns per card per day. The net length of the trajectory was 1 μs.

The same approach was employed to record the cubic-PEG-ub trajectory. In this case the initial coordinates were derived from the crystallographic structure 3N30 (ref. 17). The periodic boundary box was modelled after a single crystal unit cell, containing 48 ubiquitin molecules (equally divided between chains A and B) and 23,419 water molecules. The net length of the trajectory was 1 μs. The volume of the simulation box remains stable throughout the simulation within 0.7% of its target value (on average, there is a slight uniform contraction as described by linear factor 0.9986). Note that the statistical sampling for both chain A and chain B is the same as for the single ubiquitin chain in the MPD-ub trajectory. Finally, the rod-PEG-ub trajectory was designed based on the crystallographic coordinates 3EHV (ref. 18). The periodic boundary box was defined as a block of two crystal unit cells, containing 24 ubiquitin molecules (equally divided between chains A, B and C, which comprise the asymmetric unit) and 6,198 water molecules, for the total of 48,234 atoms.

The solution trajectory was based on the coordinate file 1UBQ^56^; this crystal structure has an excellent record in terms of interpreting the solution NMR data. The sample conditions were assumed to be pH 4.7, 300 K, matching those in the experimental study^57^. The truncated octahedral periodic boundary box contained a single ubiquitin molecule and 3,572 water molecules. The net length of the solution trajectory was 2 μs.

To calculate ^15^N–^1^H dipolar order parameters from the MPD-ub trajectory, we first superimposed all ubiquitin molecules in the periodic boundary box by applying the appropriate crystal symmetry transformations. Then ^15^N–^1^H^N^ vectors were extracted from the transformed coordinates; the vectors pertaining to each individual residue were arranged to the form of a long array (corresponding to the effective 24 μs time span). Finally, the Brüschweiler–Wright formula has been applied to these arrays to calculate *S*^2^ (ref. 58). To calculate the ^15^N relaxation rate constants, the ^15^N–^1^H dipolar correlation functions have been computed on a non-linear grid^59^. They were subsequently averaged over 24 equivalent ubiquitin molecules, as found in the crystal trajectory. The resulting curves were fitted to a combination of six exponentials and a constant. The upper bound was imposed on the fitted correlation times: they were not allowed to be longer than the length of the trajectory, that is, 1 μs. The time-modulated portion of the correlation function (that is, the six weighted exponentials) was then used to evaluate the spectral density functions and subsequently calculate the per-residue ^15^N *R*_1_ rates^60^. The same strategies were used for the other trajectories.

### XRD data collection and processing

Before being flash frozen in the cryogenic N_2_ stream on the beamline, crystals were cryoprotected with a brief soaking in a solution composed of the mother liquor complemented with 20% glycerol. Data were collected at 100 K on the ESRF ID29 (cubic-PEG-ub and rod-PEG-ub) and ID23-2 (rod-PEG-ub II) beamlines. Diffraction frames were processed with XDS^61^ and intensities were further processed with XSCALE and XDSCONV. All structures were solved using the molecular replacement technique with PHASER^62^.

### Molecular replacement and model refinement

The initial search models were ubiquitin models obtained under identical crystallization conditions, that is, 3N30 (ref. 17) and 3EHV (ref. 18) for cubic-PEG-ub and rod-PEG-ub, respectively. As expected, two and three molecules of ubiquitin were found in the molecular replacement solutions for cubic-PEG-ub and rod-PEG-ub. Rod-PEG-ub-II crystals grew in the same space group as rod-PEG-ub (P 2_1_ 2_1_ 2_1_), but with different unit cell parameters and diffracted up to 1.15 Å (Table 1). Only one ubiquitin molecule is present in the asymmetric unit of this crystal form. The refinement was conducted with PHENIX^63^. Following an initial rigid body minimization, the refinement procedure was identical for cubic-PEG-ub and rod-PEG-ub models and consisted of refinement of atomic displacement and individual isotropic B-factors. Water molecules were added to the rod-PEG-ub model using the automated water-picking option in PHENIX and were checked manually for possible close contacts with the protein. For the model of rod-PEG-ub-II, similar refinement strategy was used with the exception of anisotropic refinement of B-factors for all protein atoms, as well as water molecules. Five and six Zn^2+^ ions were modelled in cubic-PEG-ub and rod-PEG-ub coordinates, respectively, based on the presence of large positive peaks in the mFo-DFc map and taking into consideration Zn^2+^ chemical coordination. Model building was carried out with COOT^64^. For rod-PEG-ub, unexpectedly high *R*_free_ and *R*_work_ values were obtained (0.325 and 0.302, respectively). Various refinement strategies were attempted without success (for example, multiple models, TLS refinement, use of a reference model). To validate the correctness of our molecular replacement solution, we carried out a *de novo* model building, using the autobuild function of PHENIX. The initial map was computed using our experimental data and the refined ubiquitin model obtained under identical crystallization conditions (3EHV). The automated procedure was able to reconstruct 99% of the backbone and 84% of the side chains confirming the correctness of the molecular replacement solution. Cubic-PEG-ub, rod-PEG-ub and rod-PEG-ub-II have been deposited to the Protein Data Bank under the codes 4XOL, 4XOK and 4XOF, respectively.

MPD-ub crystals grew as sea urchins composed of thousands of extremely thin rods (∼100–200 × 5 × 5 μm), impossible to isolate and loop individually. We therefore performed a powder diffraction experiment, to confirm that our crystals have the same space group as the previously reported PDB entry 3ONS (which was obtained under identical conditions and comprehensively characterized by NMR). Details of the powder diffraction experiment are reported in the Supporting Information (Supplementary Fig. 11).

Stereo view images of the electron density maps are provided as Supplementary Fig. 12.

## Additional information

**Accession codes:** Coordinates and structure factors for cubic-PEG-ub, rod-PEG-ub and rod-PEG-ub-II have been deposited in the RCSB Protein Data Bank under accession codes 4XOL, 4XOK and 4XOF, respectively.

**How to cite this article:** Ma, P. *et al*. Observing the overall rocking motion of a protein in a crystal. *Nat. Commun.* 6:8361 doi: 10.1038/ncomms9361 (2015).

## Supplementary Material

Supplementary Information Supplementary Figures 1-12, Supplementary Tables 1-5 and Supplementary References

Supplementary Movie 1 Illustration of rigid-body motion of ubiquitin in MPD-ub crystals from MD simulation. The movie features a "typical" protein molecule in the sense of Figure 4. Specifically, the rocking correlation function is closest to the average rocking correlation functions, with minimum rms deviation between the two. The correlation functions classified as outliers (green curves in Figure 4) have been excluded from consideration. To generate the frames, a rigid protein structure (1UBQ) has been superimposed onto the instantaneous coordinates of the selected ubiquitin molecule in the MD trajectory (via Cα atoms in the secondary structure). The sequence of the frames, therefore, represents instantaneous orientations of the rigid-body ubiquitin molecule; the internal dynamics and translational motions have been removed. The movie covers the entire length of each trajectory, which is 1 us, and are sampled at 1 ns interval. The perspective used in this movie is identical to the one in the other two movies (obtained via the appropriate crystal symmetry transformations).

Supplementary Movie 2 Illustration of rigid-body motion of ubiquitin (chain A) in cubic-PEG-ub crystals from MD simulation. The movie features a "typical" protein molecule in the sense of Figure 4. Specifically, the rocking correlation function is closest to the average rocking correlation functions, with minimum rms deviation between the two. The correlation functions classified as outliers (green curves in Figure 4) have been excluded from consideration. To generate the frames, a rigid protein structure (1UBQ) has been superimposed onto the instantaneous coordinates of the selected ubiquitin molecule in the MD trajectory (via Cα atoms in the secondary structure). The sequence of the frames, therefore, represents instantaneous orientations of the rigid-body ubiquitin molecule; the internal dynamics and translational motions have been removed. The movie covers the entire length of each trajectory, which is 1 us, and are sampled at 1 ns interval. The perspective used in this movie is identical to the one in the other two movies (obtained via the appropriate crystal symmetry transformations).

Supplementary Movie 3 Illustration of rigid-body motion of ubiquitin (chain B) in cubic-PEG-ub crystals from MD simulation. The movie features a "typical" protein molecule in the sense of Figure 4. Specifically, the rocking correlation function is closest to the average rocking correlation functions, with minimum rms deviation between the two. The correlation functions classified as outliers (green curves in Figure 4) have been excluded from consideration. To generate the frames, a rigid protein structure (1UBQ) has been superimposed onto the instantaneous coordinates of the selected ubiquitin molecule in the MD trajectory (via Cα atoms in the secondary structure). The sequence of the frames, therefore, represents instantaneous orientations of the rigid-body ubiquitin molecule; the internal dynamics and translational motions have been removed. The movie covers the entire length of each trajectory, which is 1 us, and are sampled at 1 ns interval. The perspective used in this movie is identical to the one in the other two movies (obtained via the appropriate crystal symmetry transformations).

## Figures and Tables

**Table 1:** X-ray data collection and refinement statistics.

	**Rod-PEG-ub**	**Rod-PEG-ub II**	**Cubic-PEG-ub**
Data collection			
Space group	P 2_1_ 2_1_ 2_1_	P 2_1_ 2_1_ 2_1_	P 4_3_ 3 2
			
Cell dimensions			
*a*, *b*, *c* (Å)	43.72, 50.36, 93.46	27.94, 43.30, 50.19	104.95, 104.95, 104.95
*α*, *β*, *γ* (°)	90, 90, 90	90, 90, 90	90, 90, 90
Resolution (Å)	46.73–2.2 (2.279–2.2)	32.78–1.15 (1.191–1.15)	34.98–2.91 (3.013–2.91)
*R*_merge_	0.08323 (0.1753)	0.0609 (0.8113)	0.06642 (0.7768)
*I*/σ*I*	16.04 (7.59)	14.10 (1.93)	16.46 (2.11)
Completeness (%)	92.91 (62.00)	99.68 (98.12)	98.83 (99.34)
Redundancy	5.6 (4.9)	7.0 (6.7)	5.1 (5.1)
			
Refinement			
Resolution (Å)	46.73–2.2 (2.279–2.2)	32.78–1.15 (1.191–1.15)	34.98–2.91 (3.013–2.91)
No. of reflections	56,289 (3144)	155,489 (14390)	23,513 (2321)
*R*_work_	0.3015 (0.3538)	0.1369 (0.2230)	0.2372 (0.3805)
*R*_free_	0.3249 (0.3776)	0.1713 (0.2605)	0.2689 (0.4189)
No. of non-H atoms	1,791	789	1,191
Protein	1,703	663	1,176
Ligand/ion	6		5
Water	82	125	10
			
B-factors			
Protein	26.30	14.60	87.70
Ligand/ion	23.90	NA	87.60
Water	19.70	28.00	37.30
			
R.m.s deviations			
Bond lengths (Å)	0.007	0.010	0.005
Bond angles (°)	1.36	1.27	0.93

NA, not applicable; R.m.s., root mean squared.
