# Supplementary material for: Observing the overall rocking motion of a protein in a crystal
Source: Nat Commun. 2015 Oct 5;6:8361. doi: 10.1038/ncomms9361 (PMC4600728; doi:10.1038/ncomms9361)
Supplement: Supplementary Information — Supplementary Figures 1-12, Supplementary Tables 1-5 and Supplementary References [file ncomms9361-s1.pdf]

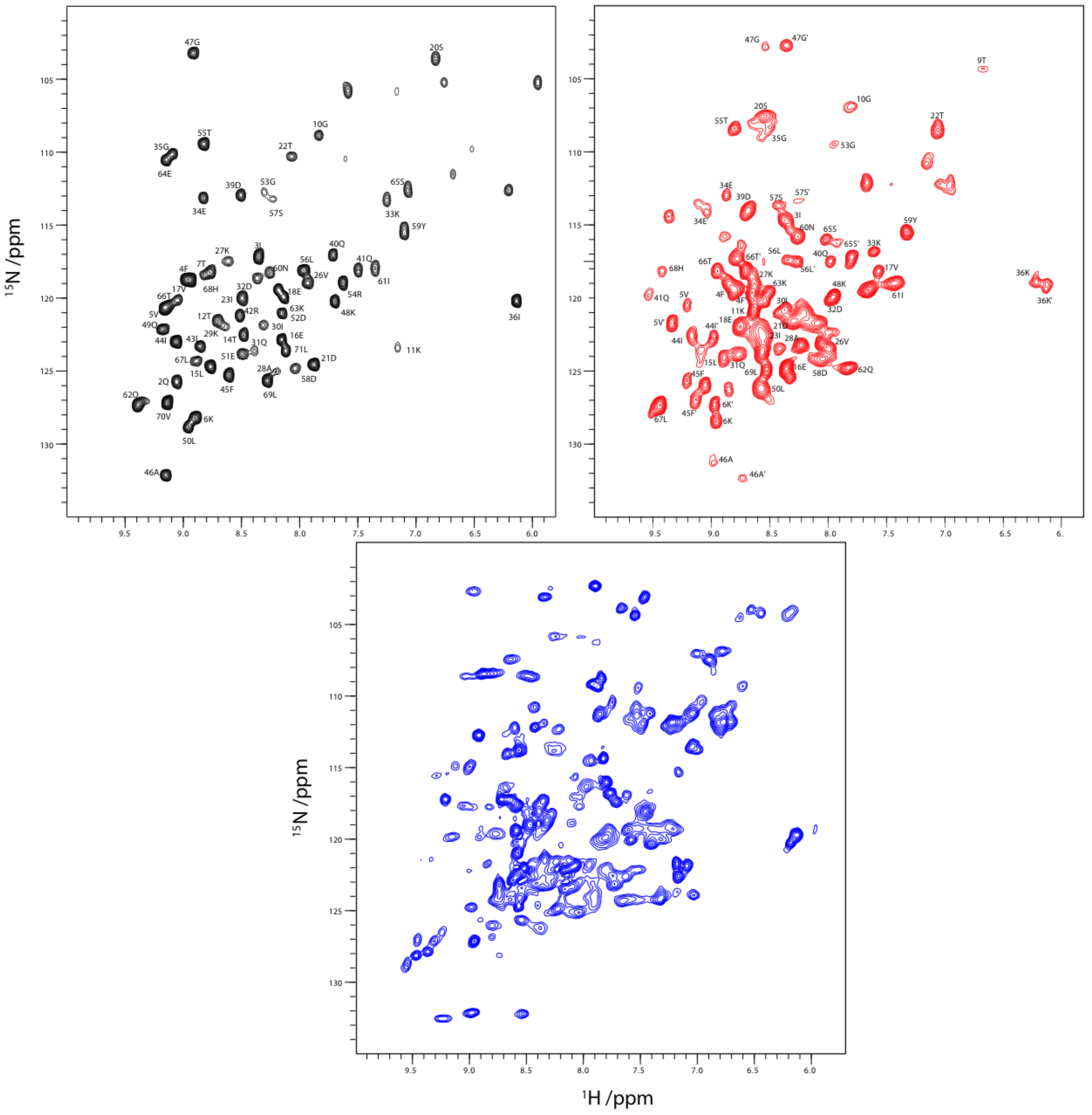

Supplementary Figure 1. Comparison of HN correlation spectra of MPD-ub (black), cubic-PEG-ub (red) and rod-PEG-ub (blue) recorded on deuterated samples with amide protons back-exchanged at a level of 50%.

The spectra were recorded at 600 MHz ^{1}H Larmor frequency with a MAS spinning frequency of 50 kHz and an effective sample temperature 300 K. Residue-specific assignments are indicated next to the spectral peaks (for the duplicate peaks in cubic-PEG-ub one of the labels is marked with a prime symbol)

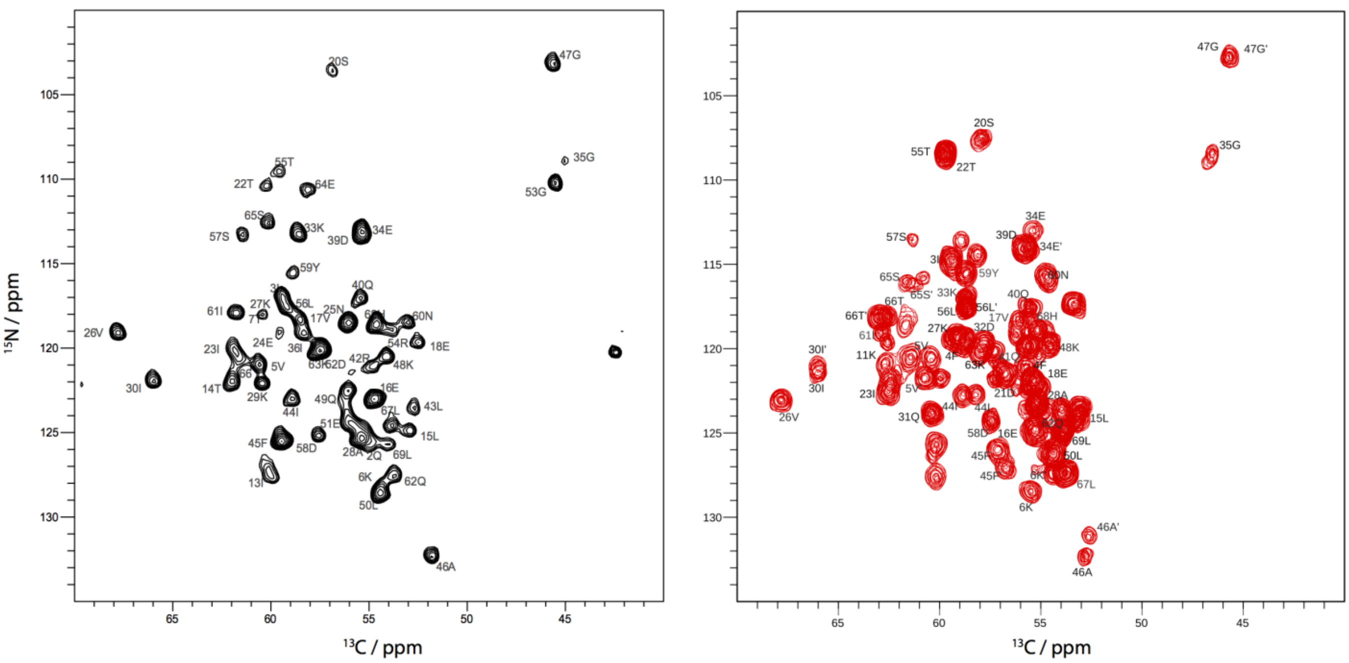

Supplementary Figure 2. N-CA correlation maps of MPD-ub (black) and cubic-PEG-ub (red) along with residue-specific assignments.

The spectrum of MPD-ub has been obtained using a ^{13}C-detected NCA correlation experiment,[1] while the spectrum of cubic-PEG-ub represents a proton-detected hCANH spectrum,[2] projected along the ^{1}H dimension. The two spectra were recorded at 600 MHz ^{1}H Larmor frequency with MAS spinning frequencies of 15 kHz (NCA, MPD-ub) and 39 kHz (hCANH, cubic-PEG-ub), respectively.

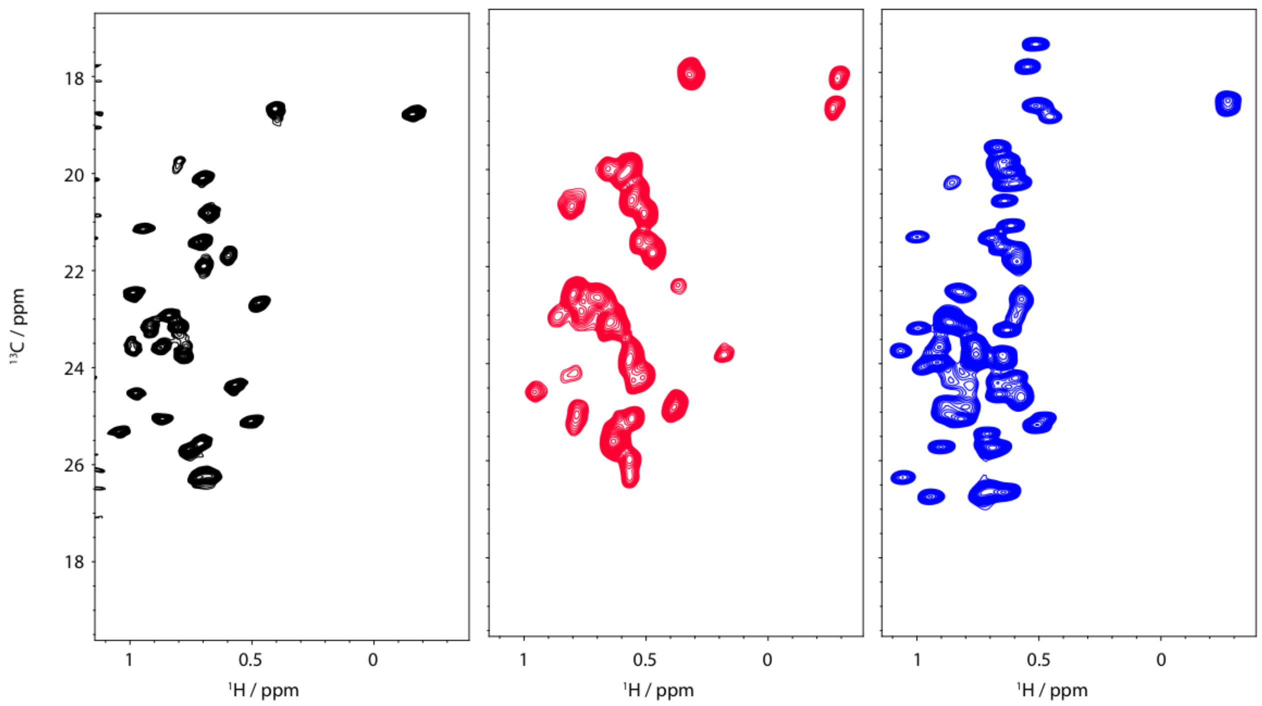

Supplementary Figure 3. Methyl ^{1}H-^{13}C correlation spectra of Val and Leu groups in MPD-ub (black), cubic-PEG-ub (red) and rod-PEG-ub (blue).

These spectra were obtained at 40 kHz MAS frequency, using samples with $^{13}CHD_2$-labeled methyl groups in Val and Leu sites. ^{2}H decoupling (3 kHz WALTZ-16) was employed during the ^{13}C evolution time, as described before[3] except for the spectrum of cubic-PEG-ub, which was recorded without deuterium decoupling, explaining at least in part the larger line widths. Site-specific assignments of methyls in MPD-ub have been previously reported.[4]

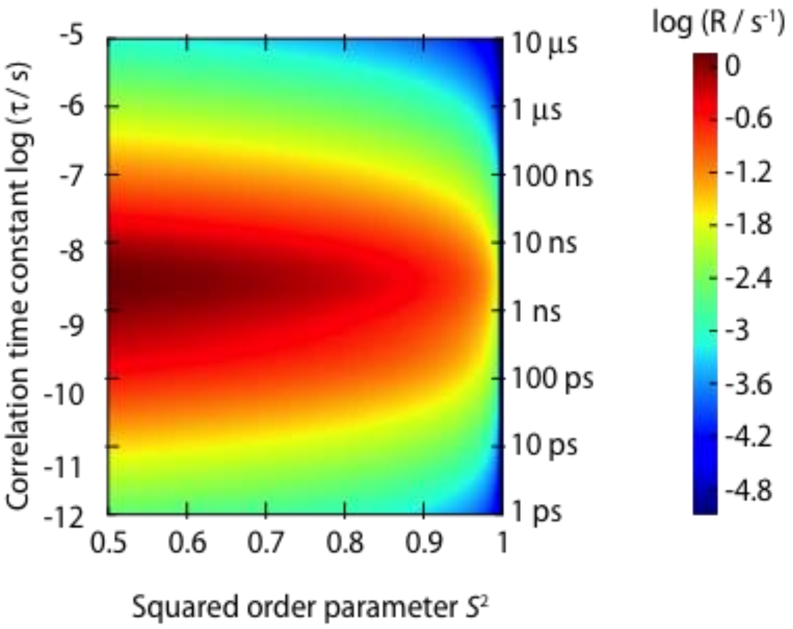

Supplementary Figure 4. The dependence of the ^{15}N R_1 relaxation rate constant on the time scale and amplitude of reorientational motion.

The R_1 rates have been computed using the model-free formalism with a single motional correlation time.[5-7] The proton-nitrogen distance was assumed to be 1.02 Å, and the anisotropy of the (presumed axially symmetric) nitrogen CSA tensor was taken to be -172 ppm. The ^1H Larmor frequency was set to 600 MHz, mimicking our experimental setup. The ^{15}N R_1 rate constants are highest for correlation times of approximately 1-10 ns. Measurable rate constants R_1>0.01 s^{-1} are produced by motions occurring on time scales from tens of picoseconds to ~100 ns, depending also on the motional amplitude. The calculations were performed ignoring the inherent multi-exponential nature of the R_1 decay that arises from orientation-dependent relaxation. We have shown previously that the error associated with this approximation is very small, generally well below the precision of experimental measurements.[7]

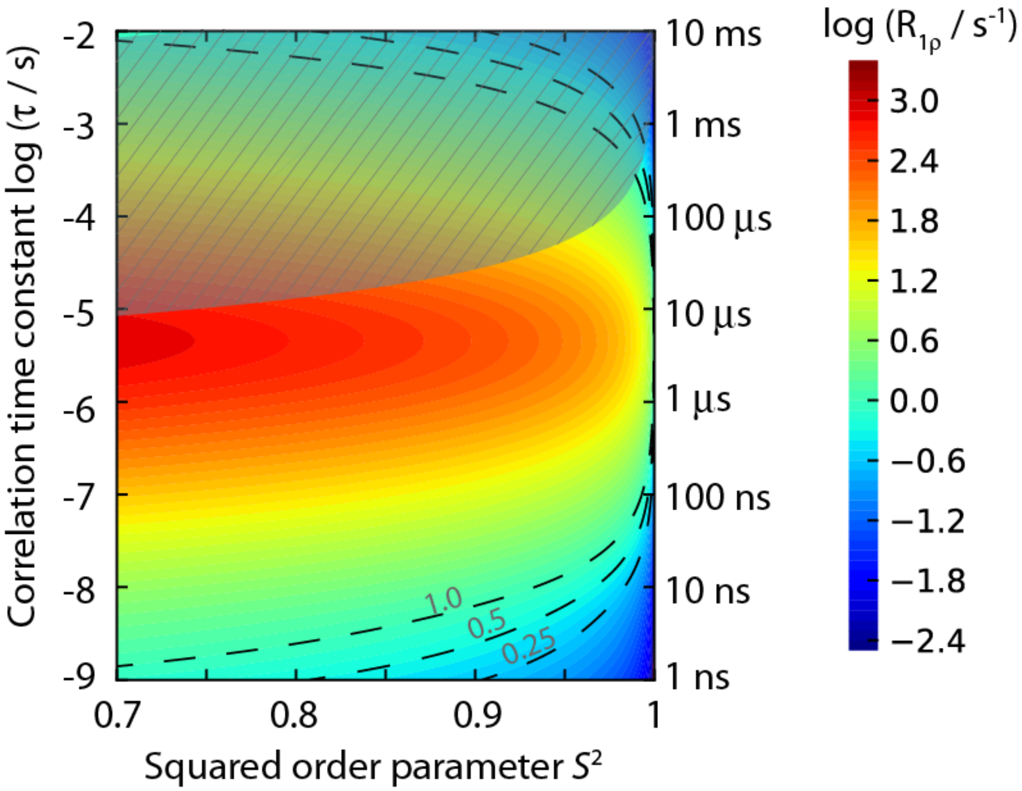

Supplementary Figure 5. The dependence of the ^{15}N $R_{1\rho}$ relaxation rate constant on the amplitude and time scale of reorientational motion.

The $R_{1\rho}$ rates have been computed using the model-free formalism with a single motional correlation time, similar to Supplementary Figure 4. Additionally, it has been assumed that $\omega_{1,15N}/2\pi$ = 15 kHz and $\omega_{MAS}/2\pi$ = 39.5 kHz, in line with our experimental setup. The calculations were conducted using the formula by Kurbanov et al., which accounts for the entry of $\omega_{1,15N}$ and ω_{MAS} frequencies into spectral densities.[8] This formula shows an appreciable difference from the standard solution-type expression for the correlation times τ exceeding ca. 1µs. Dashed lines in the plot show the relaxation rate constants close to the detection limit (determined by the maximum duration of the spin-lock period that is dictated by hardware limitations) and thus delineate the range of motional parameters to which $R_{1\rho}$ measurements are sensitive.

One should bear in mind that the formula by Kurbanov, as well as other similar results,[9] are derived from the Redfield theory. In principle, the range of validity of this formula is given by the following relationship, $2\pi(1\text{-}S)d_{NH}\tau < 0.1$, where d_{NH} is the strength of the proton-nitrogen dipolar coupling (11.5 kHz). The region where this condition is violated is shown as a grey hatched area in the plot. However, our numeric simulations suggest that Kurbanov's results remain sufficiently accurate over the broad (S^2, τ) region, see Figure 5. To explain this observation, one needs to re-analyze the conditions of validity of the Redfield treatment in the rotating frame under fast MAS conditions, as appropriate for the spin-lock experiment under consideration. Such analysis is beyond the scope of this work.

The behavior of ^{15}N $R_{1\rho}$ rate constants, as seen in the contour plot Supplementary Figure 5, can be easily rationalized. Generally, the ^{15}N $R_{1\rho}$ rate constant increases for larger motional amplitudes ($1\text{-}S^2$) and longer correlation times τ. However, when the motion becomes sufficiently slow (with τ in microsecond range) the dipolar interactions, as well as CSA interactions, are efficiently refocused by the fast magic angle spinning, as well as strong ^{15}N spin lock field. As a consequence, the $R_{1\rho}$ rate constant declines toward the upper edge of the graph.

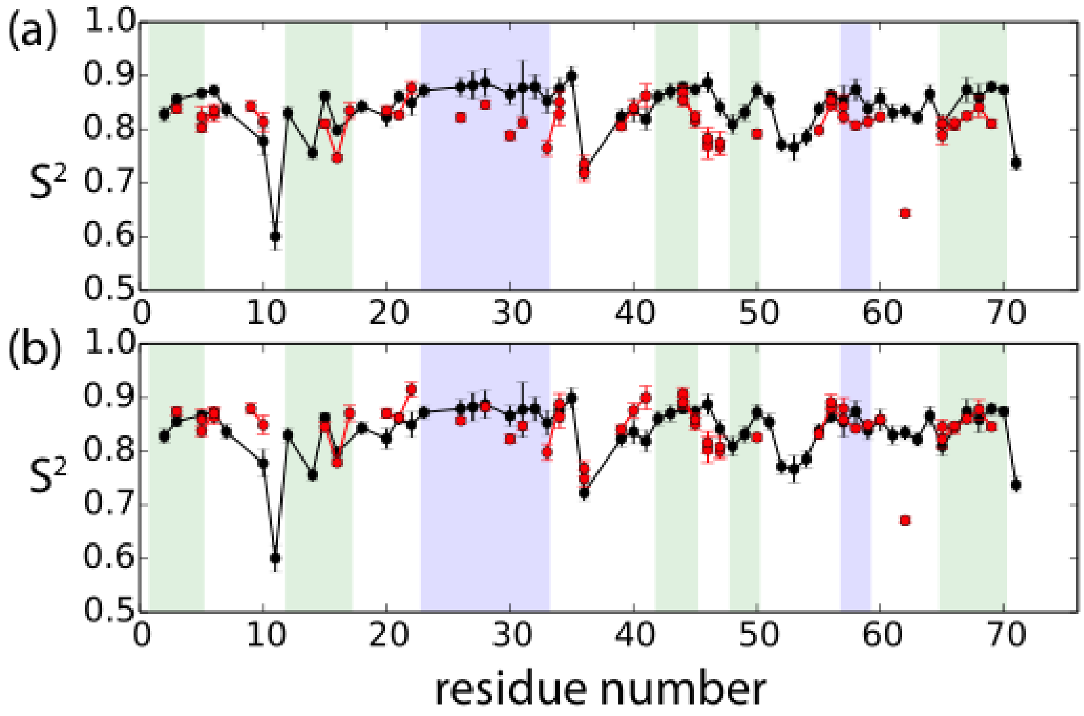

Supplementary Figure 6. Comparison of order parameters S^2 in MPD-ub (black) and cubic-PEG-ub (red).

The data in panel (a) are the experimental data shown in Figure 2 of the main text. In panel (b), the order parameters of cubic-PEG-ub have been scaled by a factor 1.04. This factor minimizes the difference between the two data sets, MPD-ub and cubic-PEG-ub, excluding residues G10 and Q62 which have clear differences in local dynamics in the two crystal forms.

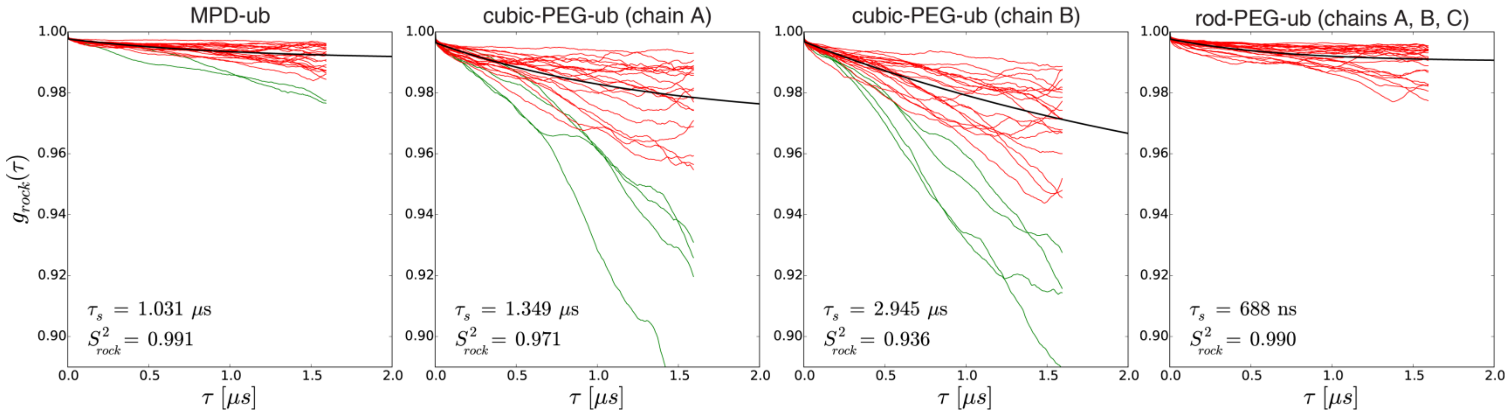

Supplementary Figure 7. Rocking motion correlation functions as extracted from the extended MD simulations of ubiquitin crystals.

To further explore the convergence properties of the MD simulations, all trajectories have been extended to 2 μs. The rocking correlation functions $g_{rock}(\tau)$ have been derived from the extended trajectories using the same procedure as described in the caption of Figure 4. The results confirm our previous findings that cubic-PEG-ub experiences intense rocking motion, while two other crystal forms are only minimally affected. At the same time, the results clearly point toward the lack of convergence. For MPD-ub, the extracted order parameter drops from 0.995 (1-μs trajectory) to 0.991 (2-μs trajectory), whereas the extracted time constant increases nine-fold from 119 ns to 1.031 μs. Similar trends are also seen for the other crystals. The extended MD results do not agree well with the experimental ^{15}N $R_{1\rho}$ data, suggesting that the MD simulations suffer from a lack of convergence and/or from "structural drift" (see main text). We have verified that the internal coordinates of ubiquitin molecules are well preserved during the simulations; therefore, it is the dynamics of the crystal lattice that is problematic. It is also instructive to discuss MD results in terms of the mean amplitude of the rocking motion. In the case of MPD-ub, the mean amplitude of the rocking motion is 4.6° if crystal structure is used as a reference. Alternatively, if one uses the average MD coordinates as a reference, the amplitude is 3.3°. The corresponding numbers for chain A in the cubic-PEG-ub trajectory are 11.5° and 6.3°. The substantial difference between the two values also points toward the lack of convergence and/or "structural drift".[10] Ultimately, our analysis confirms that MD simulations provide only a qualitative, rather than a quantitative, picture of the rocking motion in protein crystals.

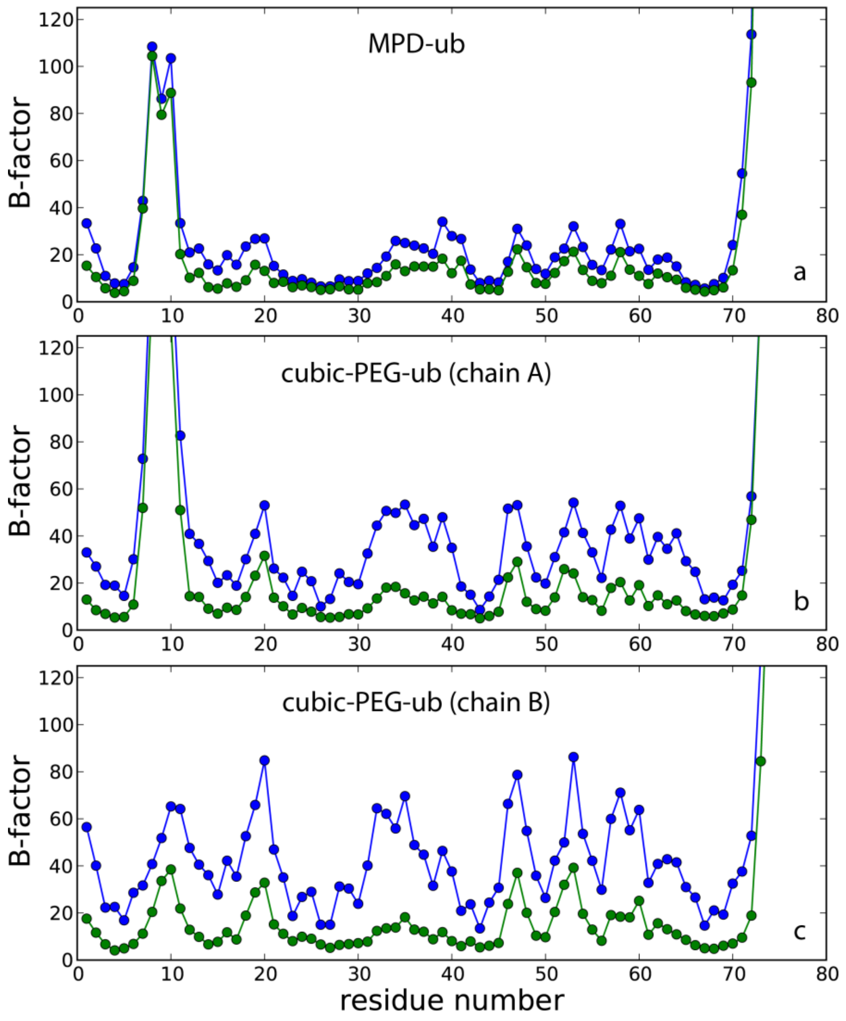

Supplementary Figure 8. Simulated B factors for C^{α} atoms in ubiquitin.

Two different protocols have been used to compute B factors on the basis of 1-μs-long crystal trajectories. In the first protocol, all protein molecules are first superimposed via the crystal symmetry transformations and then transferred to origin (through their respective centers of mass). The B factors for *i*-th atom are then evaluated as follows, $B = (8\pi^2/3)\langle(\mathbf{x}_i - \langle\mathbf{x}_i\rangle)^2\rangle$, where $\mathbf{x}_i$ is the vector of atomic coordinates and angular brackets denote averaging over all copies of the protein and all frames in the trajectory. The second protocol is different in that the copies of the protein are superimposed via the least-square fitting of the C^{α} atoms belonging to the secondary structure of the protein. Importantly, the first definition (non-aligned, blue symbols) includes the effect of re-orientational rocking dynamics, i.e. rotational fluctuations of the molecule as a whole, alongside with internal protein dynamics. In contrast, the second definition (aligned, green symbols) is confined to the motions representing internal protein dynamics. The inspection of the plot shows that rocking motion is relatively insignificant in the case of MPD-ub trajectory, but has a pronounced effect in the case of cubic-PEG simulation (especially for chain B); the results demonstrate the extent to which x-ray diffraction data deteriorate as a result of rocking dynamics in the crystal lattice. Of interest, the plot also highlights differences between chains A and B with respect to the dynamic status of the β1-β2 loop.

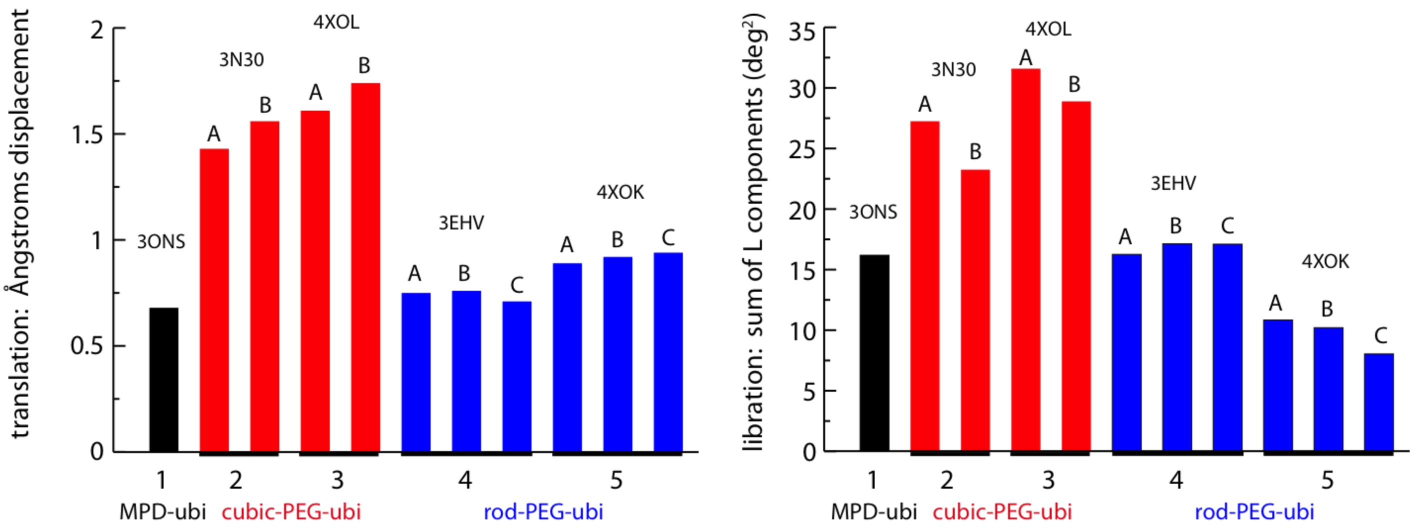

Supplementary Figure 9. TLS analysis of the three different crystal forms of ubiquitin.

The translation-libration-screw (TLS) parameters have been determined by means of the TLSMD algorithm[11,12] for three crystal forms of ubiquitin using the crystal structures available in the Protein Data Bank or obtained in this study. The PDB identifiers are marked in the plot; letters A, B and C refer to non-equivalent protein molecules in the crystal unit cells. In the TLSMD analyses each protein molecule was treated as a rigid body and has not been partitioned into segments (groups). This minimal model involves 20 unique fitting parameters, which underscores the risk of overfitting and the difficulty in interpreting the results. Note that the TLS model takes a formalized view of protein rigid-body dynamics. For instance, it is straightforward to show that a sequence of rotations with different pivot points can be described as a combination of a single rotation and a translation. It is this latter (minimalistic) description that is implemented in the TLS model, as well as other similar models such as vGNM.[13] As a consequence, the absolute values of angular fluctuations as seen by NMR (sensitive exclusively to rotation) and TLS (possibly entangled rotation/translation) are not expected to be identical.

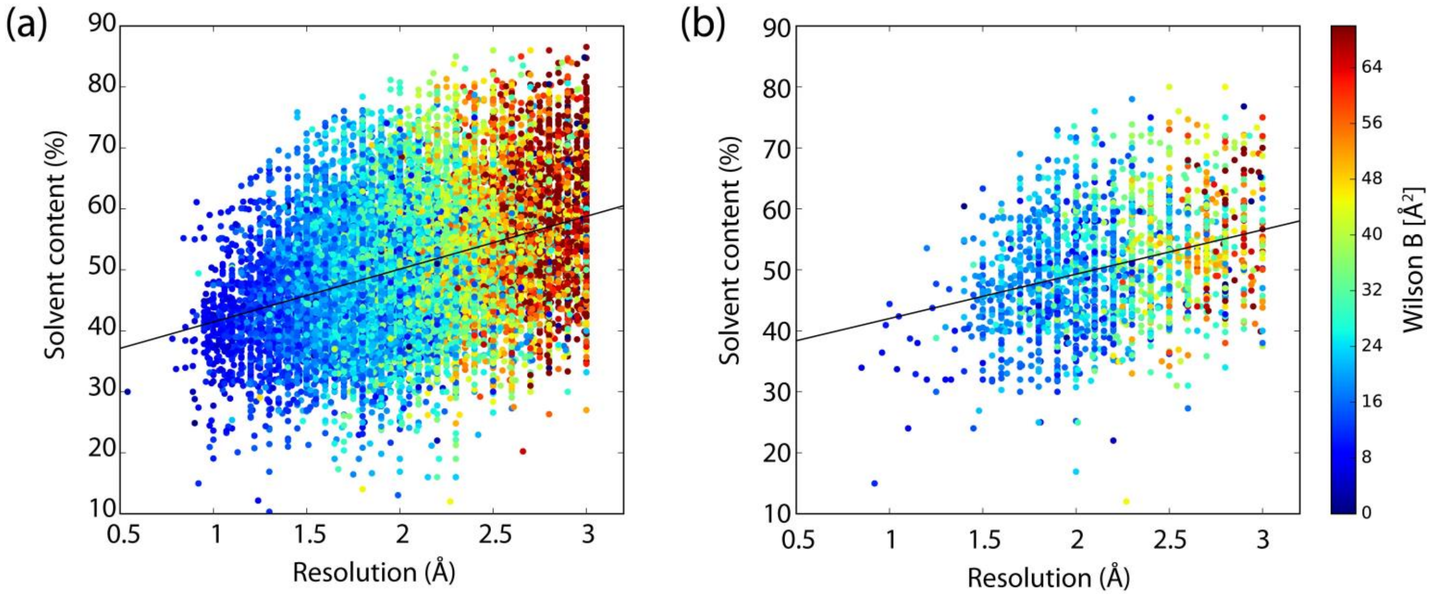

Supplementary Figure 10. Correlation between solvent content of protein crystals and resolution / Wilson B of crystallographic structures.

To generate the results in panel (a), the entire Protein Data Bank was searched for X-ray coordinate sets containing data about the solvent content in the protein crystal and an entry for the Wilson B. The plot represents a correlation between crystallographic resolution and solvent content, based on the data from 36474 collected PDB entries; the Wilson B values are color-coded. The black line represents a linear regression, according to the equation: solvent content = 8.66*resolution + 32.79 (correlation coefficient r=0.39). Panel (b) represents the subset of the data limited to those crystallographic structures that were solved at room temperature (2549 PDB entries). The black line represents a linear regression, solvent content = 7.27*resolution + 34.74 (correlation coefficient r=0.32).

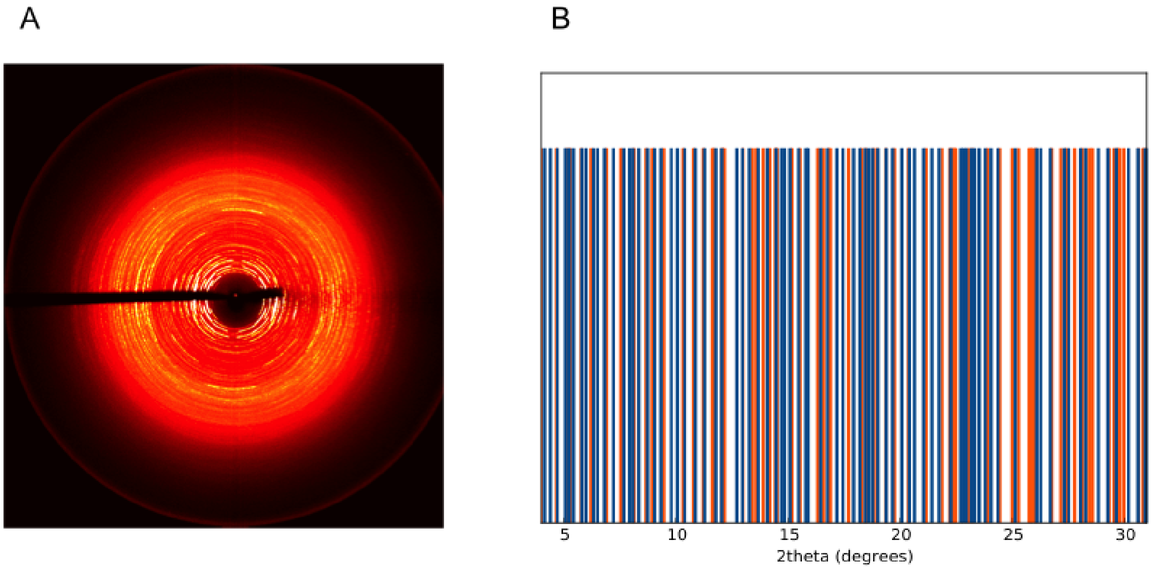

Supplementary Figure 11. Powder diffraction pattern of MPD-ub crystals.

MPD-ub crystals grew in the form of sea urchins composed of thousands of extremely thin rods (~100-200x5x5 µm), impossible to isolate and loop individually. To check whether these crystals had the same space group as those previously reported (3ONS), we looped a large number of these thin rods and collected 9 frames of 20° oscillation (180° total - 20 min exposure time per frame) using our in-house X-ray source. To generate a powder diffraction of these crystals, all frames have been summed up (shown in the left half of the figure). A simulated pattern obtained from the deposited ubiquitin model 3ONS was generated using the software powder 0.9.1 (https://pypi.python.org/pypi/powder). To compare the experimental and simulated patterns, 1D azimuthal integrations were performed as a function of 2theta using the program Fit-2D.[14] The spectral peaks obtained in this manner are represented by vertical orange bars; the simulated peaks are represented by blue bars (right half of the figure). For 2theta values ranging from 0 to 31°, 70 and 81 peaks were observed in the experimental and simulated spectra, respectively. The overall standard deviation between the 2theta values from each experimental peak and the closest simulated peak is 0.07°. The 2theta difference in peak position ranges from 0 to 0.33°. Each bar in the figure is centered at its respective 2theta value and plotted with a width corresponding to two standard deviations (0.14°). The simulated spectrum (blue) is superimposed on top of the experimental spectrum (orange) so that the agreement between the two can be judged by the number of orange bars that remain visible (out of 70).

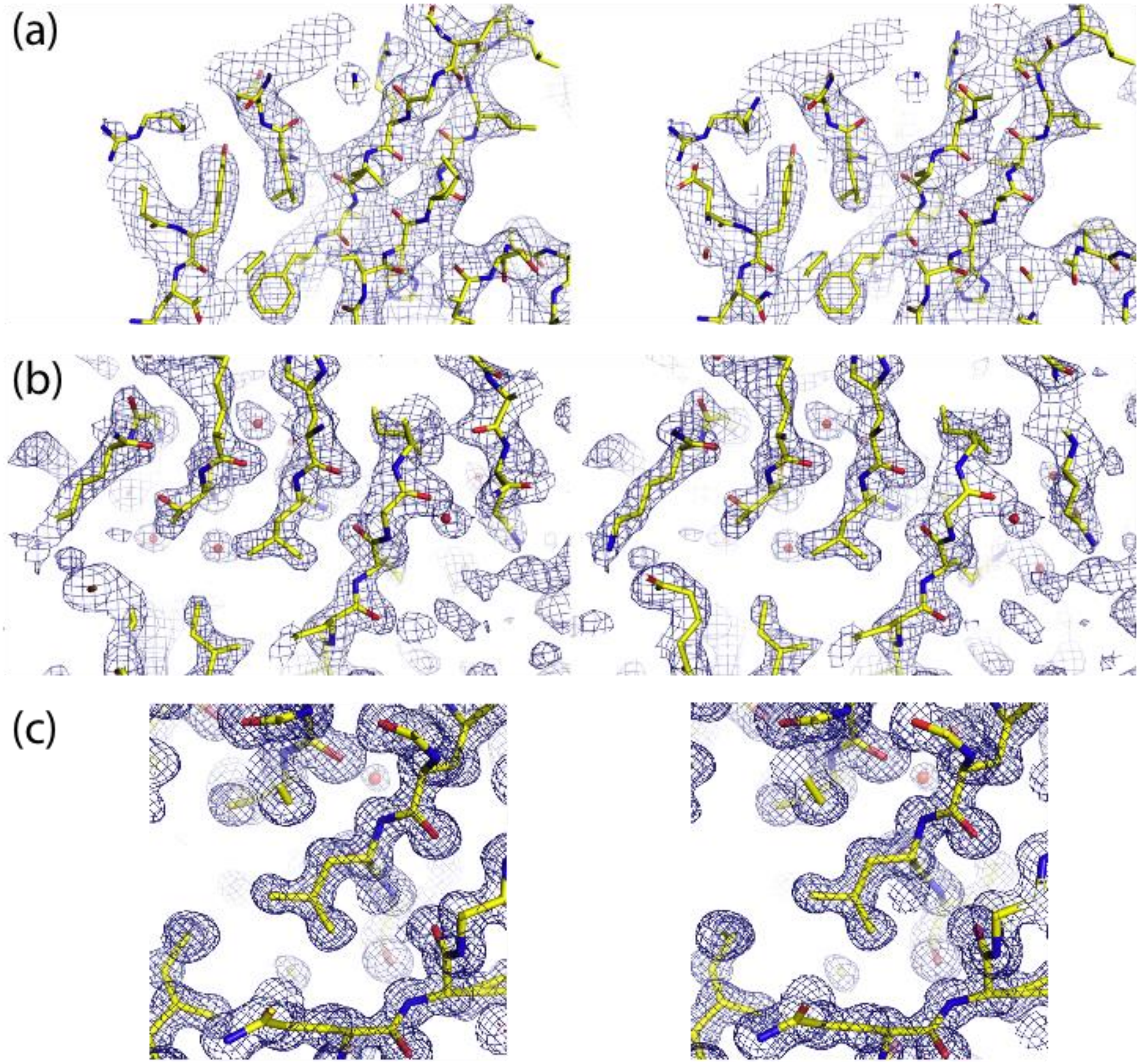

Supplementary Figure 12: Stereo view images of electron density maps.

Representative portions of the electron density maps (2mFO-DFc map, plotted at 1 σ) of (a) cubic-PEG-ub, (b) rod-PEG-ub, and (c) rod-PEG-ub-II, as determined in this study.

Supplementary Table 1. Chemical shift assignments for residues in cubic-PEG-ub.

residue number	H / ppm	N / ppm	CA / ppm	CO / ppm	CB / ppm
I3	8.29	114.44	58.94	175.79	41.70
F4	8.79	118.55	54.64	172.64	41.10
F4'	8.58	119.53	56.08	173.12	40.80
V5	9.14	120.24	59.93	175.52	34.80
V5'	9.28	121.43	61.93	174.94	33.70
K6	8.90	126.94	53.76	174.90	41.90
K6'	8.90	128.25	55.02	175.13	-
L8	-	121.80	56.30	175.00	43.85
T9	6.61	104.15	60.90	174.98	68.90
T9’	6.57	104.3	-	-	-
G10	7.76	106.68	45.90	175.37	-
K11	-	119.70			-
L15	8.83	123.84	52.59	173.86	46.50
E16	8.29	124.36	54.78	174.78	25.45
V17	7.50	117.92	55.56	175.26	32.01
S20	8.46	107.19	57.42	176.63	61.76
D21	8.23	121.26	56.22	174.97	40.23
T22	6.99	108.13	59.20	176.15	71.00
I23	8.50	122.10	62.35	179.50	34.27
V26	8.00	122.89	67.34	179.15	31.07
K27	8.58	119.30	59.11	180.70	-
K27'	8.70	119.14	59.20	177.63	33.80
A28	8.16	122.99	54.71	180.77	17.95
A28'	-	123.20	55.23	180.70	17.95
I30	8.29	120.46	56.71	176.47	66.17
Q31'	8.69	123.53	59.87	178.41	27.96
K33	7.54	117.00	58.39	177.70	34.09
K33'	7.54	116.61	58.11	177.26	34.07
E34	8.95	113.77	55.02	177.51	33.28
E34'	8.81	112.72	55.00	177.83	33.48
I36	6.15	118.57	55.67	173.10	40.67
I36	6.06	119.05	57.24	173.10	-
D39	8.61	113.79	55.31	178.15	39.85
Q40	7.90	117.18	55.23	176.68	30.34
Q41	9.46	119.52	55.34	175.68	31.88
I44	8.91	122.42	58.35	175.20	40.90
I44'	9.09	122.28	57.67	175.17	40.36
F45	9.07	126.85	56.26	175.69	43.82
F45'	8.98	125.63	56.61	175.75	44.06
A46	8.92	130.88	52.18	173.80	16.48
A46	8.66	132.10	52.36	174.44	16.68
G47	8.30	102.47	45.13	177.34	-
G47'	8.48	102.51	45.29	177.01	-
K48	7.89	119.80	54.51	174.80	34.97
Q49	-	121.90	55.34	30.28	30.28
L50	8.51	125.93	53.91	175.18	41.95
T55	8.73	108.09	59.26	175.48	72.94
L56	8.17	117.20	58.16	176.49	39.66

L56'	8.31	117.07	58.16	176.30	-
S57	8.36	113.39	60.85	180.71	62.70
S57'	8.18	113.02	60.98	180.62	-
D58	8.00	123.92	56.97	178.33	40.22
Y59	7.26	115.23	58.23	177.31	39.80
N60	8.20	115.55	54.18	174.66	37.59
Q62	7.76	124.50	53.36	174.41	32.08
K63	8.42	119.26	58.62	175.46	32.76
S65	7.95	115.65	61.15	175.10	64.96
S65'	7.84	116.01	60.75	175.10	65.03
T66	8.89	117.82	62.09	172.31	69.96
T66'	8.64	117.85	62.48	171.99	69.90
L67	9.37	126.86	53.28	173.89	44.47
L67'	9.40	127.30	53.67	175.80	44.40
H68	9.36	117.87	55.09	175.96	30.00
L69	8.45	124.68	53.41	173.37	44.43

Supplementary Table 2. Experimental dynamics data for residues in cubic-PEG-ub.

residue number	S^2	stdev S^2	R_1 / s^{-1}	stdev. R_1 / s^{-1}	$R_{1\rho}$ / s^{-1}	stdev. $R_{1\rho}$ / s^{-1}
I3	0.838	0.007	0.041	0.001	10.35	0.19
F4	-	-	0.040	0.001	12.38	0.23
F4'	-	-	0.044	0.001	7.06	0.10
V5	0.823	0.018	0.047	0.003	11.73	0.50
V5'	0.803	0.009	0.060	0.002	12.03	0.30
K6	0.828	0.013	0.038	0.002	9.93	0.24
K6'	0.835	0.012	0.028	0.002	9.32	0.25
T9	0.843	0.010	0.023	0.001	7.84	0.14
G10	0.814	0.018	0.048	0.004	16.08	0.62
L15	0.811	0.009	0.055	0.002	12.29	0.27
E16	0.747	0.004	0.090	0.001	14.67	0.16
V17	0.835	0.014	0.070	0.004	14.38	0.49
S20	0.835	0.009	0.026	0.001	11.18	0.21
D21	0.827	0.005	0.035	0.001	11.44	0.14
T22	0.877	0.013	0.024	0.001	8.43	0.17
V26	0.822	0.007	0.045	0.001	8.92	0.12
K27'	-	-	0.033	0.001	10.62	0.15
A28	0.846	0.007	0.038	0.001	8.16	0.12
I30	0.788	0.008	0.082	0.002	15.39	0.33
Q31'	0.812	0.009	0.034	0.001	10.18	0.18
K33'	0.765	0.015	0.053	0.002	11.43	0.40
E34	0.828	0.021	0.052	0.003	13.48	0.54
E34'	0.851	0.020	0.034	0.003	7.94	0.32
I36	0.718	0.015	0.070	0.001	25.61	0.88
I36’	0.736	0.015	0.062	0.001	18.03	0.57
D39	0.806	0.008	0.066	0.002	10.75	0.20
Q40	0.839	0.014	0.069	0.003	15.06	0.46
Q41	0.862	0.022	0.033	0.003	15.14	0.69
I44	0.869	0.012	0.039	0.002	13.62	0.40
I44'	0.854	0.011	0.046	0.002	10.69	0.29
F45	0.815	0.012	0.033	0.002	11.08	0.29
F45'	0.823	0.011	0.029	0.001	9.09	0.19
A46	0.770	0.024	0.077	0.006	21.06	1.26
A46	0.782	0.021	0.052	0.006	16.13	0.73
G47	0.767	0.014	0.106	0.005	16.70	0.56
G47'	0.774	0.021	0.091	0.006	35.40	2.25
L50	0.792	0.007	0.050	0.001	13.49	0.20
T55	0.798	0.011	0.037	0.002	14.60	0.41
L56	0.844	0.011	0.028	0.001	9.84	0.23
L56'	0.854	0.014	0.024	0.002	8.80	0.26
S57	0.823	0.014	0.046	0.002	13.08	0.42
S57'	0.843	0.021	0.036	0.004	10.49	0.65
D58	0.807	0.008	0.047	0.001	13.72	0.22
Y59	0.814	0.011	0.053	0.002	10.12	0.19
N60	0.823	0.009	0.066	0.002	13.00	0.30
Q62	0.643	0.005	0.200	0.005	22.25	0.47
K63	-	-	0.090	0.003	9.99	0.23

S65	0.811	0.013	0.066	0.003	14.63	0.51
S65'	0.788	0.017	0.076	0.004	9.61	0.41
T66	0.807	0.010	0.039	0.001	14.01	0.31
T66'	0.812	0.006	0.040	0.001	8.34	0.11
L67	0.825	0.006	0.036	0.001	12.14	0.18
H68	0.841	0.019	0.026	0.002	11.24	0.43
L69	0.811	0.007	0.046	0.001	10.80	0.19

Supplementary Table 3. Simulated dynamics parameters for residues in MPD-ub.

Residue	R_1 / s^{-1}	S^2	B-factor (CA)
01-MET	-	-	33.35
02-GLN	4.97E-02	0.864	22.68
03-ILE	2.21E-02	0.915	10.98
04-PHE	1.69E-02	0.897	7.81
05-VAL	1.90E-02	0.910	7.55
06-LYS	4.19E-02	0.857	14.64
07-THR	9.12E-02	0.793	42.89
08-LEU	1.48E-01	0.609	108.40
09-THR	2.02E-01	0.526	86.25
10-GLY	3.14E-01	0.535	103.49
11-LYS	3.20E-01	0.363	33.41
12-THR	1.43E-01	0.670	20.95
13-ILE	7.69E-02	0.820	22.63
14-THR	3.26E-02	0.845	15.88
15-LEU	3.75E-02	0.902	13.33
16-GLH	3.27E-02	0.862	19.80
17-VAL	1.88E-02	0.894	15.74
18-GLH	2.97E-02	0.903	23.48
19-PRO	-	-	26.72
20-SER	3.80E-02	0.857	26.91
21-ASP	1.77E-02	0.876	15.22
22-THR	1.74E-02	0.875	11.58
23-ILE	1.16E-02	0.898	8.86
24-GLH	1.09E-02	0.937	9.59
25-ASN	1.19E-02	0.906	8.09
26-VAL	8.74E-03	0.916	6.54
27-LYS	7.95E-03	0.921	6.55
28-ALA	1.06E-02	0.931	9.51
29-LYS	1.25E-02	0.910	8.77
30-ILE	1.20E-02	0.889	8.85
31-GLN	1.29E-02	0.926	12.06
32-ASP	1.19E-02	0.932	14.40
33-LYS	6.98E-02	0.797	19.22
34-GLH	5.73E-02	0.842	25.86
35-GLY	3.47E-02	0.863	24.98
36-ILE	7.64E-02	0.747	23.88
37-PRO	-	-	22.69
38-PRO	-	-	20.38
39-ASH	5.65E-02	0.799	34.03
40-GLN	7.94E-02	0.780	27.92
41-GLN	7.32E-02	0.705	26.71
42-ARG	3.82E-02	0.752	13.69
43-LEU	3.38E-02	0.874	7.97
44-ILE	2.04E-02	0.894	8.99
45-PHE	1.77E-02	0.931	8.23
46-ALA	3.16E-02	0.885	17.02
47-GLY	6.02E-02	0.843	31.00

48-LYS	6.47E-02	0.799	23.96
49-GLN	5.40E-02	0.862	13.91
50-LEU	5.30E-02	0.877	11.89
51-GLH	5.37E-02	0.827	18.90
52-ASP	4.99E-02	0.838	22.58
53-GLY	5.12E-02	0.773	32.06
54-ARG	1.09E-01	0.611	23.27
55-THR	3.25E-02	0.873	15.68
56-LEU	1.23E-02	0.905	13.44
57-SER	2.19E-02	0.890	22.19
58-ASP	1.62E-02	0.896	33.11
59-TYR	4.13E-02	0.810	21.41
60-ASN	2.53E-02	0.873	22.52
61-ILE	2.84E-02	0.903	13.58
62-GLN	4.61E-02	0.835	17.95
63-LYS	2.84E-02	0.877	18.79
64-GLH	2.39E-02	0.912	15.02
65-SER	2.53E-02	0.844	8.24
66-THR	4.79E-02	0.844	7.18
67-LEU	2.75E-02	0.865	5.65
68-HIP	3.52E-02	0.863	7.51
69-LEU	3.30E-02	0.835	10.13
70-VAL	2.67E-02	0.881	24.11
71-LEU	4.55E-02	0.816	54.52
72-ARG	9.11E-02	0.526	113.61
73-LEU	2.69E-01	0.231	347.00
74-ARG	2.43E-01	0.121	565.63
75-GLY	3.76E-01	0.085	876.73
76-GLY	4.08E-01	0.024	1261.78

Supplementary Table 4. Simulated dynamics parameters for residues in cubic-PEG-ub.

	Chain A			Chain B		
Residue	R_1 / s^{-1}	S^2	B-factor (CA)	R_1 / s^{-1}	S^2	B-factor (CA)
01-MET	-	-	31.5233	-	-	41.4761
02-GLN	6.13E-02	0.82275	24.2505	7.10E-02	0.82047	29.2801
03-ILE	3.05E-02	0.88639	15.1881	2.86E-02	0.88046	18.4444
04-PHE	2.00E-02	0.88345	15.3888	2.39E-02	0.878	18.4266
05-VAL	2.06E-02	0.88671	12.3525	1.92E-02	0.8927	12.9107
06-LYS	3.60E-02	0.85732	23.6263	2.27E-02	0.88175	20.9958
07-THR	8.27E-02	0.69088	59.6866	6.10E-02	0.80792	25.4824
08-LEU	1.03E-01	0.63532	124.8366	5.35E-02	0.83467	34.0328
09-THR	1.46E-01	0.55503	153.1926	3.72E-02	0.85087	42.9468
10-GLY	1.91E-01	0.49449	144.0438	6.95E-02	0.78305	51.5961
11-LYS	2.62E-01	0.26763	71.2487	1.44E-01	0.62187	45.6729
12-THR	1.65E-01	0.48996	32.7206	1.43E-01	0.69633	35.4957
13-ILE	8.68E-02	0.77144	31.4886	4.14E-02	0.82863	31.0308
14-THR	4.71E-02	0.82209	25.664	3.18E-02	0.83966	28.0134
15-LEU	3.28E-02	0.87691	18.6532	6.39E-02	0.84687	22.19
16-GLU	5.27E-02	0.83332	23.5489	4.71E-02	0.82976	30.7315
17-VAL	3.56E-02	0.87229	19.9192	3.69E-02	0.85607	28.3315
18-GLU	6.54E-02	0.84344	30.2792	7.38E-02	0.80925	42.3553
19-PRO	-	-	34.5965	-	-	55.3063
20-SER	6.18E-02	0.79707	39.8892	7.33E-02	0.74957	62.2947
21-ASP	4.47E-02	0.83306	23.3925	4.17E-02	0.82524	35.0666
22-THR	5.85E-02	0.81358	17.2851	6.96E-02	0.77666	26.5032
23-ILE	2.34E-02	0.88891	11.5058	3.01E-02	0.87713	15.0876
24-GLU	2.23E-02	0.89115	19.527	2.54E-02	0.89056	20.8235
25-ASN	2.04E-02	0.87765	17.7409	2.02E-02	0.88623	21.959
26-VAL	1.92E-02	0.88987	8.909	1.97E-02	0.88107	12.3898
27-LYS	1.43E-02	0.90504	10.5866	1.41E-02	0.90481	12.5344
28-ALA	1.26E-02	0.89288	18.0325	1.37E-02	0.89071	23.8793
29-LYS	2.47E-02	0.88773	16.936	1.74E-02	0.8831	23.0324
30-ILE	2.58E-02	0.87056	15.953	2.01E-02	0.87389	17.9852
31-GLN	2.18E-02	0.88774	24.2668	1.94E-02	0.89483	30.8603
32-ASP	3.88E-02	0.84502	33.8875	2.71E-02	0.869	47.2597
33-LYS	1.23E-01	0.69409	40.036	8.97E-02	0.73175	45.2606
34-GLU	6.66E-02	0.76577	35.6224	4.80E-02	0.83024	37.6313
35-GLY	5.14E-02	0.79876	36.3909	3.14E-02	0.83479	46.6374
36-ILE	6.91E-02	0.69207	29.4379	8.36E-02	0.68895	33.7822
37-PRO	-	-	31.7119	-	-	34.8352
38-PRO	-	-	24.495	-	-	25.3542
39-ASP	3.43E-02	0.84431	33.8214	2.89E-02	0.85849	37.2078
40-GLN	5.51E-02	0.81557	22.7894	4.29E-02	0.8433	30.5144
41-GLN	8.00E-02	0.72375	14.8474	5.67E-02	0.81071	16.2694
42-ARG	3.69E-02	0.86565	13.5922	2.46E-02	0.86248	18.8181
43-LEU	3.27E-02	0.86704	8.3194	4.01E-02	0.82503	11.1171
44-ILE	1.94E-02	0.89131	13.647	1.82E-02	0.88653	18.5318
45-PHE	1.53E-02	0.89812	19.2836	2.03E-02	0.88577	23.6212
46-ALA	2.62E-02	0.86029	46.1056	5.27E-02	0.81692	52.9721
47-GLY	3.21E-02	0.8162	49.2147	8.85E-02	0.75385	66.2035

48-LYS	3.58E-02	0.80915	34.1643	6.99E-02	0.77505	44.0347
49-GLN	2.74E-02	0.8525	20.0568	7.00E-02	0.77164	27.9374
50-LEU	1.59E-02	0.87771	17.2073	6.02E-02	0.81346	21.2637
51-GLU	6.10E-02	0.79995	27.2144	7.84E-02	0.73444	33.6324
52-ASP	4.40E-02	0.81649	32.8159	8.04E-02	0.71455	35.7584
53-GLY	4.79E-02	0.72474	43.9599	1.44E-01	0.57864	68.0221
54-ARG	4.28E-02	0.72938	30.7318	9.56E-02	0.66754	37.4751
55-THR	2.95E-02	0.82899	23.3128	4.60E-02	0.80136	26.7717
56-LEU	2.27E-02	0.90445	17.4055	2.38E-02	0.8653	18.3438
57-SER	3.34E-02	0.86107	34.1545	4.02E-02	0.84189	38.6298
58-ASP	2.21E-02	0.85371	39.8032	2.84E-02	0.84101	47.0109
59-TYR	3.69E-02	0.82596	30.3404	5.80E-02	0.79309	33.7013
60-ASN	2.83E-02	0.84993	37.5063	3.79E-02	0.84095	41.6575
61-ILE	3.52E-02	0.84627	23.6467	5.06E-02	0.83946	23.2304
62-GLN	6.82E-02	0.76966	31.3965	8.73E-02	0.7411	31.6784
63-LYS	4.52E-02	0.85498	27.9058	5.85E-02	0.85137	34.5467
64-GLU	3.68E-02	0.86215	30.4718	2.97E-02	0.88488	35.1482
65-SER	4.11E-02	0.78074	21.232	3.76E-02	0.82838	26.5264
66-THR	2.75E-02	0.83716	17.955	3.35E-02	0.8459	22.35
67-LEU	2.95E-02	0.85848	11.1555	2.79E-02	0.84922	12.2744
68-HIE	3.29E-02	0.8604	12.8482	3.37E-02	0.86529	16.212
69-LEU	3.49E-02	0.83588	11.9124	6.88E-02	0.77076	14.5973
70-VAL	3.83E-02	0.85723	19.6337	1.99E-02	0.87869	23.9043
71-LEU	3.93E-02	0.84939	20.4449	3.45E-02	0.84642	31.0678
72-ARG	1.04E-01	0.63998	43.6067	6.39E-02	0.75177	42.4968
73-LEU	1.95E-01	0.33383	119.1994	2.35E-01	0.36238	102.1798
74-ARG	3.12E-01	0.2603	270.2161	2.04E-01	0.32169	202.602
75-GLY	6.92E-01	0.11683	572.9459	4.98E-01	0.15971	375.1609
76-GLY	7.94E-01	0.0538	977.1049	7.00E-01	0.03469	747.3563

Supplementary Table 5. Simulated dynamics parameters for ubiquitin in solution.

Residue	S^2	Residue	S^2	Residue	S^2
02-GLN	0.85431	32-ASP	0.87781	63-LYS	0.86664
03-ILE	0.90046	33-LYS	0.75911	64-GLU	0.89908
04-PHE	0.90326	34-GLU	0.82158	65-SER	0.837
05-VAL	0.90897	35-GLY	0.8587	66-THR	0.86948
06-LYS	0.88642	36-ILE	0.73644	67-LEU	0.87569
07-THR	0.84433	39-ASP	0.8694	68-HIE	0.89496
08-LEU	0.64351	40-GLN	0.8444	69-LEU	0.83326
09-THR	0.62301	41-GLN	0.77457	70-VAL	0.89016
10-GLY	0.6558	42-ARG	0.89747	71-LEU	0.85201
11-LYS	0.55556	43-LEU	0.87559	72-ARG	0.65192
12-THR	0.75754	44-ILE	0.89869	73-LEU	0.27662
13-ILE	0.81252	45-PHE	0.91565	74-ARG	0.17354
14-THR	0.84591	46-ALA	0.86071	75-GLY	0.0248
15-LEU	0.88796	47-GLY	0.81933	76-GLY	0.00574
16-GLU	0.83982	48-LYS	0.7876		
17-VAL	0.88315	49-GLN	0.83405		
18-GLU	0.86948	50-LEU	0.83811		
20-SER	0.83836	51-GLU	0.80254		
21-ASP	0.88108	52-ASP	0.8116		
22-THR	0.84301	53-GLY	0.69383		
23-ILE	0.90396	54-ARG	0.71143		
24-GLU	0.90552	55-THR	0.84506		
25-ASN	0.89442	56-LEU	0.90893		
26-VAL	0.90227	57-SER	0.87162		
27-LYS	0.92289	58-ASP	0.87024		
28-ALA	0.90781	59-TYR	0.82066		
29-LYS	0.89831	60-ASN	0.8583		
30-ILE	0.89076	61-ILE	0.85509		
31-GLN	0.90297	62-GLN	0.7686		

Supplementary References

1. Baldus, M., Petkova, A. T., Herzfeld, J. & Griffin, R. G. Cross polarization in the tilted frame: assignment and spectral simplification in heteronuclear spin systems. *Mol Phys* **95,** 1197–1207 (1998).
2. Knight, M. J. *et al.* Fast resonance assignment and fold determination of human superoxide dismutase by high-resolution proton-detected solid-state MAS NMR spectroscopy. *Angew Chem Int. Ed. Engl.* **50,** 11697–11701 (2011).
3. Huber, M. *et al.* A supplementary coil for 2H decoupling with commercial HCN MAS probes. *J Magn Reson* **214,** 76–80 (2012).
4. Huber, M. *et al.* A proton-detected 4D solid-state NMR experiment for protein structure determination. *ChemPhysChem* **12,** 915–918 (2011).
5. Lipari, G. & Szabo, A. Model-free approach to the interpretation of nuclear magnetic-resonance relaxation in macromolecules: 1. Theory and range of validity. *J Am Chem Soc* **104,** 4546–4559 (1982).
6. Chevelkov, V., Fink, U. & Reif, B. Quantitative analysis of backbone motion in proteins using MAS solid-state NMR spectroscopy. *J Biomol NMR* **45,** 197–206 (2009).
7. Schanda, P., Meier, B. H. & Ernst, M. Quantitative analysis of protein backbone dynamics in microcrystalline ubiquitin by solid-state NMR spectroscopy. *J Am Chem Soc* **132,** 15957–15967 (2010).
8. Kurbanov, R., Zinkevich, T. & Krushelnitsky, A. The nuclear magnetic resonance relaxation data analysis in solids: general $R_1/R_{1\rho}$ equations and the model-free approach. *J Chem Phys* **135,** 184104 (2011).
9. Fares, C., Qian, J. & Davis, J. H. Magic angle spinning and static oriented sample NMR studies of the relaxation in the rotating frame of membrane peptides. *J Chem Phys* **122,** 194908 (2005).
10. Janowski, P. A., Liu, C., Deckman, J. & Case, D. A. Molecular dynamics simulation of triclinic lysozyme in a crystal lattice. *Protein Sci* (2015). doi:10.1002/pro.2713
11. Painter, J. & Merritt, E. A. Optimal description of a protein structure in terms of multiple groups undergoing TLS motion. *Acta Crystallogr. D.* **62,** 439–450 (2006).
12. Painter, J. & Merritt, E. A. TLSMD web server for the generation of multi-group TLS models. *J. Appl. Crystallogr.* (2006).
13. Song, G. & Jernigan, R. L. vGNM: a better model for understanding the dynamics of proteins in crystals. *J Mol Biol* **369,** 880–893 (2007).
14. Hammersley, A. P. ESRF Internal Report, ESRF98HA01T, FIT2D V9.129 Reference Manual V3.1 (1998).
